# Long‐Chain Fatty Acid Oxidation Disorder Genes: A Comprehensive Genetic Database of LC‐FAOD Variants, Genotypes, and Phenotypes

**DOI:** 10.1155/humu/6864813

**Published:** 2026-07-07

**Authors:** Heather Richbourg, Vanessa Rangel Miller, Omid Khazaie Japalaghi, Moeenaldeen AlSayed, Peter R. Baker, Sean Daugherty, Tali Ekstein, Sarah C. Grünert, Mark J. Kiel, Aneal Khan, Hironori Kobayashi, Lawrence Korngut, Stephanie A. Monteleone, Ida Vanessa D. Schwartz, Nicole Miller, Jerry Vockley

**Affiliations:** ^1^ Ultragenyx Pharmaceutical Inc., Novato, California, USA; ^2^ College of Medicine, Al-Faisal University, Riyadh, Riyadh Province, Saudi Arabia, alfaisal.edu; ^3^ Anschutz Medical Campus, University of Colorado, Aurora, Colorado, USA, colorado.edu; ^4^ Labcorp, San Francisco, California, USA; ^5^ Children′s Hospital, Department of Pediatrics, Adolescent Medicine and Neonatology, Faculty of Medicine, Freiburg University Hospital, Freiburg, Baden-Württemberg, Germany; ^6^ Genomenon, Ann Arbor, Michigan, USA; ^7^ M.A.G.I.C. (Metabolics and Genetics in Canada) Clinic Ltd., Calgary, Alberta, Canada; ^8^ Laboratories Division, Shimane University Hospital, Izumo, Shimane Prefecture, Japan, shimane-u.ac.jp; ^9^ Department of Clinical Neurosciences, University of Calgary, Calgary, Alberta, Canada, ucalgary.ca; ^10^ Hospital de Clinicas, Porto Alegre, Rio Grande do Sul, Brazil; ^11^ Division of Medical Genetics and Center for Rare Disease Therapy, University of Pittsburgh, Pittsburgh, Pennsylvania, USA, pitt.edu

**Keywords:** cardiomyopathy, hypoglycemia, locus-specific database, long-chain fatty acid oxidation disorders (LC-FAODs), rhabdomyolysis

## Abstract

Long‐chain fatty acid oxidation disorders (LC‐FAODs) are characterized by the inability to metabolize long‐chain fatty acids. Serious clinical manifestations occur, including cardiomyopathy, hypoglycemia, rhabdomyolysis, and liver failure. Confirming a diagnosis with genetic testing is complicated by the rarity of the disorders, genetic and phenotypic heterogeneity, and the high frequency of variants of uncertain significance. A new locus‐specific database for variants in the six genes associated with LC‐FAOD was established to collect and disseminate information about disease‐associated variants in *ACADVL*, *CPT1A*, *CPT2*, *HADHA*, *HADHB*, and *SLC25A20*. The database integrates data from a systematic literature review and a sponsored gene panel program with associated clinical and biochemical data. The database was reviewed and curated by an expert panel and stored in MongoDB and MySQL. As of March 2025 (literature review cutoff), the database reports 6947 variants from 4188 individuals with ≥ 1 variant in an LC‐FAOD gene. *ACADVL* variants are the most common (40%), followed by *HADHA* (25%), *CPT2* (21%), *CPT1A* and *HADHB* (5% each), and *SLC25A20* (4%). Associated phenotypes are reported for 1496 individuals, newborn screening results for 2589, and enzyme activity assays for 499 individuals. Severe outcomes (cardiomyopathy < 1 year or death at any age) are reported for 219 individuals with ≥ 2 P/LP LC‐FAOD gene variants, and the most common genotype among them is homozygosity for the LCHAD variant, *HADHA* p.Glu510Gln (*n* = 48/219). The LC‐FAOD gene database is a comprehensive archive of variants, genotypes, and phenotypes associated with this important group of FAODs. It is open to the greater scientific and LC‐FAOD communities through a public website.

## 1. Introduction

**Figure 1:**
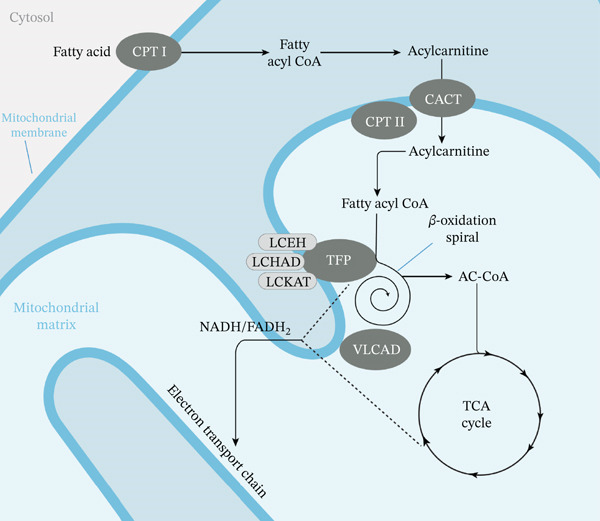
Long‐chain fatty acid oxidation pathway highlighting the enzymes impacted in LC‐FAOD. Activated long‐chain fatty acyl‐CoA esters are transported across the mitochondrial membrane via the carnitine shuttle [6]. In this three‐step process, Carnitine Palmitoyltransferase 1 (CPT I) covalently links carnitine to long‐chain fatty acyl‐CoA. The resulting acylcarnitine intermediate is transported across the membrane by carnitine/acylcarnitine translocase (CACT) in exchange for a free carnitine molecule, and finally, carnitine is removed from the acyl‐CoA by Carnitine Palmitoyltransferase 2 (CPT II) [6]. Inside mitochondria, most long‐chain fatty acyl‐CoAs are used for energy production via *β*‐oxidation. Very‐long‐chain acyl‐CoA dehydrogenase (VLCAD) controls the initial dehydrogenation step in the spiral, producing a long‐chain enoyl‐CoA that then undergoes hydration, a second dehydrogenation step, and finally thiolytic cleavage to produce one molecule of acyl‐CoA and a fatty acyl‐CoA shortened by two carbons [4]. These final three reactions for long‐chain substrates are performed by the three enzymatic activities of the mitochondrial trifunctional protein (TFP): long‐chain enoyl‐CoA hydratase (LCEH), long‐chain (S)‐3‐hydroxyacyl‐CoA dehydrogenase (LCHAD), and long‐chain 3‐ketoacyl‐CoA thiolase (LCKAT) [4, 7].

Long‐chain fatty acid oxidation disorders (LC‐FAODs) are rare, autosomal recessive diseases caused by abnormalities in nuclear genes that encode mitochondrial proteins essential for the oxidation of fatty acids of 12–18 carbons in chain length [1–4]. LC‐FAODs are comprised of deficiencies in six different protein/protein activities: Carnitine Palmitoyltransferase 1 (CPT I), carnitine/acylcarnitine translocase (CACT), Carnitine Palmitoyltransferase 2 (CPT II), very‐long‐chain acyl‐CoA dehydrogenase (VLCAD), long‐chain 3‐hydroxyacyl‐CoA dehydrogenase (LCHAD), and mitochondrial trifunctional protein (TFP), which are part of the carnitine shuttle system or the mitochondrial beta‐oxidation pathway (Figure 1) [3, 5]. In patients with LC‐FAOD, there can be a deficit in energy production and accumulation of toxic metabolites such as long‐chain acylcarnitines.

Common clinical features in LC‐FAOD are hypoglycemia, cardiomyopathy, and recurrent rhabdomyolysis. TFP and LCHAD deficiency have neuropathy and retinopathy as unique features (Table 1). Patients with LC‐FAOD require lifelong medical care from metabolic disease specialists. Management involves avoidance of prolonged fasting, sometimes restriction of long‐chain fats in the diet, and early intervention such as intravenous dextrose during illness, to avoid hypoglycemia and clinical decompensation [8]. One medication is approved in Brazil, Canada, Kuwait, Mexico, and the United States for treatment of LC‐FAODs (triheptanoin). Clinical outcomes for patients were poor prior to the introduction of newborn screening (NBS), with up to 70% mortality by age 10 years; while survival is now higher with NBS, early death is still common, and morbidity is nearly ubiquitous [9]. Clinical manifestations of each LC‐FAOD type, along with the associated gene and protein, are summarized in Table 1.

**Table 1:** LC‐FAOD genes, types, and differentiating features.

Gene(s)/location	Disorder type	Diagnostic acylcarnitine profile	Common clinical presentations
*CPT1A/*11q13.3 (OMIM 600528)	Carnitine Palmitoyltransferase I (CPT I) deficiency	C0/(C16 + C18)↑ ([10]; [11]). Can be missed in plasma [12]	Recurrent episodes of fasting‐induced hypoketotic hypoglycemia and risk of liver failure. Heterozygous females can develop fatty liver of pregnancy when carrying an affected fetus [13]
*SLC25A20*/3p21.31 (OMIM 613698)	Carnitine/acylcarnitine translocase (CACT) deficiency	(C16 + C18:1)/C2↑ [14]	Generally, the most severe and earliest onset form of LC‐FAOD. Mortality remains high even with NBS; surviving patients can experience arrhythmia and hyperammonemia, profound developmental delay, and seizures even with treatment ([15]; [16]; [17])
*CPT2/*1p32.3 (OMIM 600650)	Carnitine Palmitoyltransferase II (CPT II) deficiency	(C16 + C18:1)/C2↑ [14]. Can be missed in dried blood spot [12]	Congenital malformations, including dysmorphic features, renal cysts, and intracerebral and intrahepatic calcifications, can occur in lethal neonatal form or a mild form with mainly muscular symptoms, which is due to the common variant p.Ser113Leu [18]. Recurrent rhabdomyolytic decompensations, exercise intolerance [19]
*ACADVL/*17p13.1 (OMIM 609575)	Very long‐chain acyl‐CoA dehydrogenase (VLCAD) deficiency	C14:1↑, C14:1/C2↑ ([11]; [20])	Cardiomyopathy at any age, exercise intolerance, and recurrent rhabdomyolytic decompensations [20]
*HADHA/*2p23.3 (OMIM 600890)	Long‐chain 3‐hydroxy‐acyl‐CoA dehydrogenase (LCHAD) deficiency	C16OH↑, C18OH↑ [21]	Peripheral neuropathy and pigmentary retinopathy can develop; increased risk for female heterozygous carriers to develop hemolysis, elevated liver enzymes, low‐platelet (HELLP) syndrome, and acute fatty liver of pregnancy (AFLP) ([22]; [1])
*HADHA*, *HADHB*/2p23.3 (OMIM 600890, 143450)	Trifunctional protein (TFP) deficiency	C16OH↑, C18OH↑ [21]	Severe peripheral neuropathy: Pigmentary retinopathy is also present, though less dramatic than in LCHAD deficiency ([23]; [1])

CPT I is a 773 amino acid protein encoded by one of three tissue‐specific gene isoforms [24]. To date, disease‐causing variants have only been identified in *CPT1A*, encoding the liver isoform [5]. Disease‐causing variants in *CPT1A* occur across the entire gene and are highly heterogeneous, with some notable exceptions, all of which are missense variants. In North America, c.1436C > T (p.Pro479Leu), reported with a milder phenotype, is found across Inuit, Alaskan Native, Canadian First Nations, and Hutterite populations [1, 8, 9, 25, 26], and c.2129G > A (p.Gly710Glu) is found in North American Hutterites [27, 28]. Two additional recurrent variants, c.2122A > C (p.Ser708Arg) and c.1364A > C (p.Lys455Thr), are found in the Niuean and Finnish populations, respectively [29, 30].

CACT is a 301 amino acid protein encoded by the *SLC25A20* gene [31, 32]. Disease‐causing variants that have been reported are mostly found in single families with few recurring variants. Notable recurrent variants include the splicing variant c.199‐10 T > G, found in patients from East Asia; the missense variant c.713A > G (p.Gln238Arg), found in patients of Arabic descent [1, 9, 33]; the missense variant c.82G > T (p. Gly28Cys), found in Pakistani and Indian populations [29, 34, 35]; and the frameshift variant c.270del (p.Phe91Leufs∗38), found in Turkish populations [36, 37].

CPT II is a 658 amino acid protein encoded by the *CPT2* gene [38]. Disease‐causing variants in *CPT2* are reported across the entire gene with a common missense variant p.Ser113Leu, found in ~60% of patients with later‐onset myopathic disease presentation, and the c.1239_1240del; c.1342 T > C (p.Lys414Thrfs∗7; Phe448Leu) haplotype found in Ashkenazi Jewish populations [18, 9, 39].

VLCAD is a 655 amino acid protein encoded by the *ACADVL* gene [40]. Disease‐causing variants in *ACADVL* are distributed across the gene and show a high degree of molecular and clinical heterogeneity except for the highly prevalent c.848 T > C (p.Val283Ala) variant associated with a mild phenotype [9, 41, 42] and the notable c.709 T > C (p.Cys237Arg) variant associated with cardiac disease [20, 9].

Mitochondrial TFP (also referred to as MTP) is a heterotetramer of two *α* and two *β* subunits encoded by *HADHA* and *HADHB* [43]. Variants in either gene can destabilize the entire complex and disrupt all three enzymatic activities [1, 5, 8]. Isolated LCHAD deficiency has been reported in patients with disease‐associated variants in *HADHA*, predominantly c.1528G > C (p.Glu510Gln), which lies in the catalytic site of the LCHAD domain [22, 9, 44].

LC‐FAOD can initially be suspected based on NBS results or clinical presentation but must be confirmed by other testing modalities such as plasma acylcarnitine profiling by MS/MS, molecular genetic analysis, and enzyme activity assays (faodinfocus.com/diagnosis/, Baker and Burton et al. [45]). Since acylcarnitine testing to confirm positive/abnormal NBS results can be complicated by the nutritional and clinical state after birth, molecular testing is increasingly used to confirm an LC‐FAOD diagnosis, highlighting the need for a robust description of LC‐FAOD‐associated variants.

With a growing body of molecular information on LC‐FAOD arising from clinical testing, we have established a new locus‐specific database for variants in the six genes associated with LC‐FAOD to collect and disseminate information to the scientific and LC‐FAOD communities about disease‐causing variants. The database is available online at https://www.rarediseasegenes.com/lc-faod/. The website includes variant tables and a genotype table with searchable, customizable data displays for frequency, biochemical findings, phenotypes, and geographies. Users can contribute variant data to the LC‐FAOD gene database using an established variant submission process, subject to review and curation by a committee of medical and scientific experts prior to inclusion.

## 2. Methods

### 2.1. Assembling the Database

The LC‐FAOD gene variant database combines data from three sources: (1) a comprehensive, systematic review of all published medical literature through March 2025 using the Mastermind Genomic Search Engine [46]; (2) data collected through July 1, 2025, in no‐charge sponsored genetic testing programs for LC‐FAODs initiated by Ultragenyx Pharmaceutical, Inc.; and (3) data collected in Ultragenyx clinical programs and integrated with the database in January 2025. The genetic testing programs provide multigene next‐generation sequencing (NGS) panels performed by commercial genetic testing laboratories for genes known to be involved in the mitochondrial fatty acid oxidation pathway to eligible patients with a clinical diagnosis or suspicion of LC‐FAOD based on NBS results or clinical signs/symptoms.

Variants were annotated with clinical and biochemical phenotypes extracted from literature reports, clinical testing submission forms, and clinical trial records. Note that clinical trial records did not include classifications for reported variants. Data from all sources were assembled and stored in a MongoDB database and manually reviewed to eliminate duplicates, ensure accuracy, and standardize nomenclature. Additionally, variants were evaluated through Ensembl′s variant effect predictor (VEP) to provide genomic coordinates, SIFT and PolyPhen predictions, and protein effect classifications. Additional classifications were made to standardize variant categories across the different data sources, including geographic and phenotype data. See Table 1 for a list of genes and typical clinical phenotypes.

### 2.2. Patients and Privacy

Variant and genotype data are presented in aggregate. To protect patient privacy in this set of ultrarare diseases, phenotype and geographical data are presented in aggregate for variants and genotypes reported in at least five individuals. All individuals who provided samples for genetic testing through a gene panel program or clinical trial consented to having their deidentified genetic and clinical information published.

### 2.3. Variant Nomenclature and Classification

The nomenclature used in the LC‐FAOD database follows the Human Genome Variant Society (HGVS) guidelines. Sequence variants were described using NCBI reference sequence NM_000018.3 or NM_000018.4 (*ACADVL*), NM_001876.3 or NM_001876.4 (*CPT1A*), NM_000098.3 (*CPT2*), NM_000182.4 or NM_000182.5 (*HADHA*), NM_000183.2 or NM_000183.3 (*HADHB*), and NM_000387.6 (*SLC25A20*) for each gene and NP_000009.1 (ACADVL), NP_001027017.1 (CPT I), NP_000089.1 (CPT II), NP_000173.2 (HADHA), NP_000174.1 (HADHB), and NP_000378.1 (SLC25A20) for the corresponding proteins. Variants sourced from the literature review were assigned a predicted variant classification based on curations from literature, as well as data taken from multiple external databases for computational predictions (PolyPhen, SIFT, MutationTaster, and dbscSNV) and population frequencies (gnomAD) and classified according to the joint consensus from the American College of Medical Genetics and Genomics and the Association for Molecular Pathology [47]. Variants from the sponsored testing program were interpreted using laboratory‐established variant classification frameworks ([47]; Nykamp et al. [48]). Variants common between the two datasets were assigned the laboratory‐provided classification. The database primarily includes variants with some evidence of being disease‐related (classified as pathogenic [P], likely pathogenic [LP], or variant of uncertain significance [VUS]). In a few instances, variants with a predicted classification of benign or likely benign were retained in the database after additional data led to their reclassification from VUS to benign or likely benign.

### 2.4. Analysis

When available, biochemical lab values and reported clinical phenotypes were used to examine genotype/phenotype correlations. Biochemical data were interpreted according to the testing laboratories′ normal values; data reported without associated normal values were excluded from the analysis. Analysis of acylcarnitine profiles focused on the diagnostic species for the suspected disease, as outlined in Table 1. Geography is reported based on the location of the testing lab or the location of the study for variants originating from publications.

## 3. Results

### 3.1. LC‐FAOD Genetic Database Summary

As of March 2025, the LC‐FAOD database reports 6947 total variants in six LC‐FAOD genes from 4188 individuals harboring one or more LC‐FAOD gene variants. Variants were most commonly reported in *ACADVL* (2752/40%), followed by *HADHA* (1745/25%), *CPT2* (1484/21%), *HADHB* (330/5%), *CPT1A* (329/5%), and *SLC25A20* (307/4%). These represent single or repeated occurrences of 1217 variants (*ACADVL*, 546/45%; *CPT2*, 203/17%; *HADHA*, 150/12%; *HADHB*, 130/11%; *CPT1A*, 114/9%; and *SLC25A20*, 74/6%) (Table S1). A majority of these variants (*n* = 746, 61%) were unique to the literature dataset, 20% (*n* = 248) were unique to the gene testing programs, 2% (*n* = 19) were unique to the clinical programs, and 17% (*n* = 204) were identified in multiple sources. The most common variant classifications in the database are P (*n* = 499, 41%), followed by VUS (*n* = 451, 37%) and LP (*n* = 236, 19%). Classifications were not available for 22 variants (2%) that were reported among 55 patients in the dataset from clinical trials. The remaining < 1% of variants are classified as benign (*n* = 3) or likely benign (*n* = 4) or have conflicting predicted classifications (*n* = 2).

**Figure 2:**
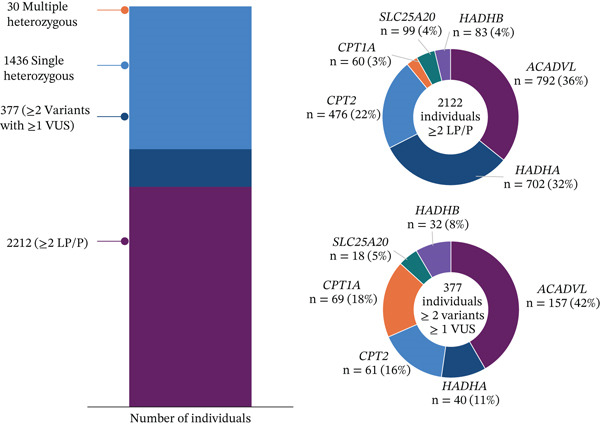
LC‐FAOD genotypes among 4055 ^∗^ individuals in the database.  ^∗^Not graphed: 133 individuals (41 who had 1 P/LP/VUS variant and 1 unknown/benign or conflicting variant; 37who had only benign/likely‐benign or conflicting variants, and 55 from clinical trial data with no variant classification reported). P: pathogenic; LP: likely pathogenic; VUS: variant of uncertain significance. Single heterozygous: 1 P/LP/VUS variant identified; multiple heterozygous: heterozygous for P/LP/VUS variants in 2 or more genes.

About half of the individuals represented in the database (*n* = 2212, 53%) have genotypes with ≥ 2 P/LP variants in an LC‐FAOD gene combined by gene as follows: *ACADVL*, 792 (36%); *HADHA*, 702 (32%); *CPT2*, 476 (22%); *SLC25A20*, 99 (4%); *HADHB*, 83 (4%); and *CPT1A*, 60 (3%) (Figure 2, Table S2). Three hundred seventy‐seven individuals in the database (9%) have two variants in an LC‐FAOD gene with ≥ 1 VUS (Table S2). Another 1436 individuals have one P/LP/VUS variant (single heterozygous), and 41 have one P/LP/VUS and one benign/conflicting variant (Figure 2, Table S3). An additional 30 individuals have P/LP/VUS variants reported in two or more LC‐FAOD genes (multiple heterozygous) (Figure 2, Table S4). Eight hundred thirty‐four unique genotypes and 646 unique variants were identified among individuals with ≥ 2 P/LP variants. Individuals with ≥ 2 variants with one or more VUSs report a total of 310 unique genotypes and 426 unique variants.

**Figure 3:**
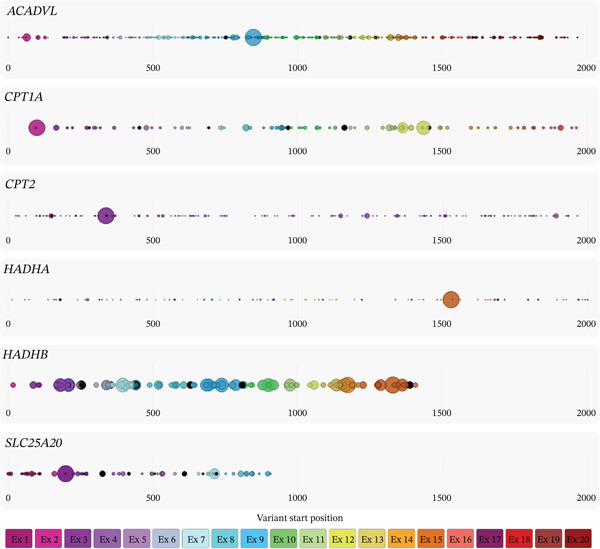
Distribution of variants across the 6 LC‐FAOD genes. Each circle indicates the position of a variant within the respective LC‐FAOD gene. Circle size indicates the relative frequency of that variant within each gene; scale differs by gene and is proportional to the number of variants in each. Variants that begin within exons are displayed in a color associated with the exon number; variants that begin within introns are displayed as black circles. Genes are shown at varying scales based on gene size, as indicated by the nucleotide positions marked below each graph.

Variants are heterogeneous and distributed across the genes (Figure 3); no common recurring variants were identified beyond those previously reported, and almost half (*n* = 596) were unique variants, each found in a single individual. The LC‐FAOD gene locus–specific database includes 853 variants reported in ClinVar, plus an additional 331 variants that are not found in ClinVar, including 248 variants that were only identified in LC‐FAOD genetic testing programs (Table S5). The remaining 26 variants could not be accurately compared as they lack precise cDNA boundaries (e.g., some copy number variants [CNVs] and variants reported with amino acid change only).

**Figure 4:**
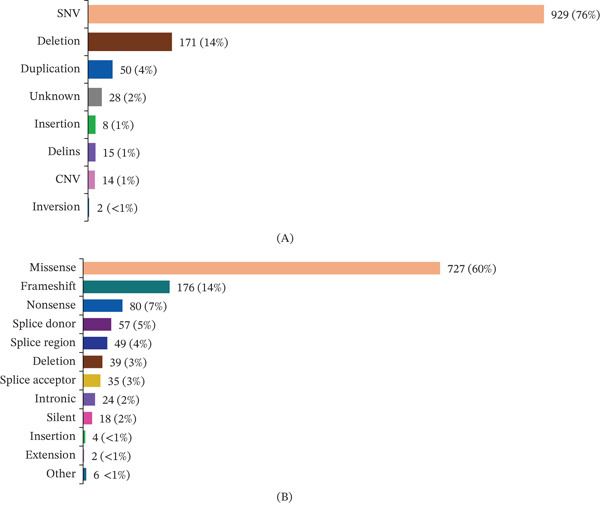
Distribution of 1217 LC‐FAOD gene variants by (A) variant type and (B) protein effect. CNV: copy number variant (copy number gain or copy number loss of > 100 bp); SNV: single‐nucleotide variant. (A) More than three‐quarters of variants were single‐nucleotide variations (SNVs), deletions comprised 14% of variants, and all other types represent < 5% of variants. Unknown: 28 variants were documented by amino acid change only, and the cDNA variant type is unknown. (B) The most common protein effect of variants was missense (60%), followed by frameshift (14%), nonsense (7%), and splice donor (5%); the remaining categories each represent < 5% of variants. “Other” includes protein‐altering (*n* = 2), duplication (*n* = 1), UTR variant (*n* = 1), and variant with unknown protein effect (*n* = 2).

Single‐nucleotide variants (SNVs) are the most common type of unique variant in the database (*n* = 929, 76%) (Figure 4A, Table 2). Fourteen percent of the unique LC‐FAOD variants are small deletions (< 100 bp, *n* = 171); duplications (< 100 bp) occur at ~4% frequency (*n* = 50), while CNVs (> 100 bp) and deletion/insertions (< 100 bp) each represent ~1% of the unique LC‐FAOD variants (Figure 4A). The most common protein effect of LC‐FAOD variants is missense (*n* = 727, 14%), followed by frameshift (*n* = 176, 14%), nonsense (*n* = 80, 7%), and other effects (≤ 5% each). Among the SNVs, ~20% cause protein effects that have clear functional consequences (nonsense and splicing). However, most SNVs cause missense protein changes (*n* = 701/929, 75% of SNVs).

**Table 2:** Distribution of 1217 variants by variant type and protein effect for each LC‐FAOD gene.

**Variant type (cDNA)**	***CPT1A***	***SLC25A20***	***CPT2***	***ACADVL***	***HADHA***	***HADHB***

SNV	101 (89%)	56 (76%)	147 (72%)	410 (75%)	108 (72%)	107 (82%)
Deletion	10 (9%)	9 (12%)	33 (16%)	82 (15%)	25 (17%)	12 (9%)
Duplication	1 (< 1%)	3 (4%)	8 (4%)	27 (5%)	8 (5%)	3 (2%)
Unknown	0 (0%)	0 (0%)	10 (5%)	16 (3%)	1 (1%)	1 (1%)
Delins	0 (0%)	1 (1%)	4 (2%)	4 (1%)	5 (3%)	1 (1%)
Insertion	1 (< 1%)	1 (1%)	1 (< 1%)	3 (1%)	1 (1%)	1 (1%)
CNV	1 (< 1%)	4 (8%)	0 (0%)	2 (< 1%)	2 (1%)	5 (4%)
Inversion	0 (0%)	0 (0%)	0 (0%)	2 (< 1%)	0 (0%)	0 (0%)
Total	114	74	203	546	150	130

**Variant protein effect**	***CPT1A***	***SLC25A20***	***CPT2***	***ACADVL***	***HADHA***	***HADHB***

Missense	76 (67%)	36 (49%)	132 (65%)	334 (61%)	68 (45%)	81 (62%)
Frameshift	9 (8%)	9 (12%)	35 (17%)	83 (15%)	28 (19%)	12 (9%)
Nonsense	10 (9%)	9 (12%)	14 (7%)	24 (4%)	13 (9%)	10 (8%)
Splice donor	4 (4%)	4 (5%)	2 (1%)	27 (5%)	18 (12%)	2 (2%)
Splice region	7 (6%)	4 (5%)	4 (2%)	21 (4%)	7 (5%)	6 (5%)
Deletion	1 (1%)	5 (7%)	4 (2%)	17 (3%)	4 (3%)	8 (6%)
Splice acceptor	1 (1%)	2 (3%)	5 (2%)	13 (2%)	8 (5%)	6 (5%)
Intronic	2 (2%)	4 (5%)	1 (< 1%)	10 (2%)	2 (1%)	5 (4%)
Silent	4 (4%)	1 (1%)	2 (1%)	10 (2%)	1 (1%)	0 (0%)
Insertion	0 (0%)	0 (0%)	1 (< 1%)	3 (1%)	0 (0%)	0 (0%)
Extension	0 (0%)	0 (0%)	0 (0%)	2 (< 1%)	0	0 (0%)
Other	0 (0%)	0 (0%)	3 (1%)	2 (< 1%)	1 (1%)	0 (0%)
Total	114	74	203	546	150	130

*Note:* Unknown: 21 variants were documented by amino acid change only, and the cDNA variant type is unknown. Other protein effects were one protein‐altering and one unknown protein effect (*CPT2*), one duplication (*HADHA*), one UTR variant, and one unknown protein effect (*ACADVL*).

Abbreviations: CNV, copy number variant (> 100 bp); SNV, single‐nucleotide variant.

Individuals in the database have a broad geographical distribution encompassing North America, South America, Europe, the Middle East, Asia, and Oceania (Figure S1). At the time of diagnosis, most individuals with ≥ 2 P/LP LC‐FAOD variants were under 1 year or 1–12 years old (*n* = 1013, 79%). This frequency continues to decrease with age, and only 42 (3%) of these individuals were over 40 at diagnosis (*n* = 1282 individuals with ≥ 2 P/LP variants and age reported, Figure S2).

Abnormal NBS results for LC‐FAOD were reported for 1370 individuals, of whom 657 (48%) have ≥ 2 P/LP variants, 154 (11%) have ≥ 2 variants with ≥ 1 VUS, 516 (38%) have only one variant (single heterozygous), and 18 (1%) have single variants in two different LC‐FAOD genes (Figure S3). Negative/false‐negative NBS results were reported for 10 individuals with ≥ 2 P/LP LC‐FAOD gene variants (6 CPT II, 3 LCHAD/TFP, and 1 TFP, Figure S3).

**Figure 5:**
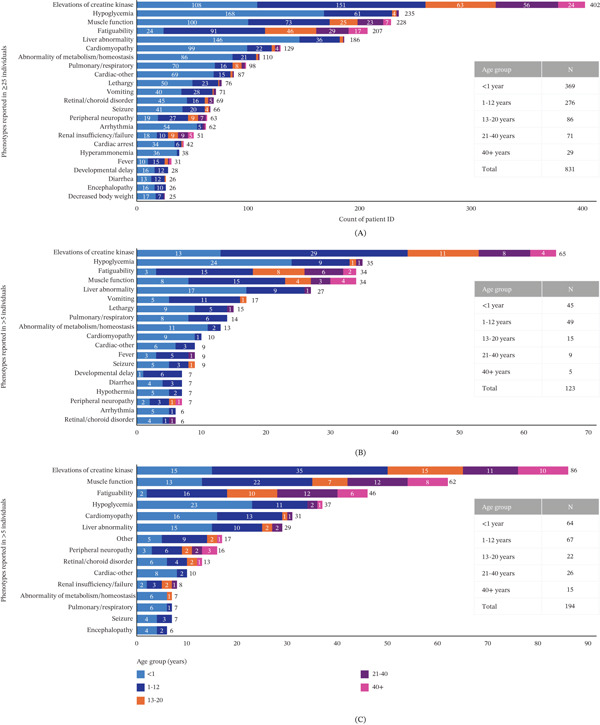
LC‐FAOD phenotypes by age group. One thousand, one hundred and ninety‐seven of 4188 individuals had phenotypes and age reported. Cardiac‐other includes structural heart defects not expected to be related to disease. Muscle phenotypes were grouped into three categories: elevations of creatine kinase (e.g., rhabdomyolysis), muscle fatiguability/pain, and abnormal muscle function/strength. (A) Individuals with ≥ 2 P/LP variants (*n* = 831). The graph shows phenotypes reported in ≥ 25 individuals. (B) Individuals with ≥ 2 variants including ≥ 1 VUS (*n* = 123). The graph shows phenotypes reported in > 5 individuals. (C) Individuals with 1 P/LP/VUS variant (*n* = 194). The graph shows phenotypes reported in > 5 individuals.

A clinical diagnosis consistent with LC‐FAOD was reported in 87% (*n* = 2911/3356) of individuals reported in the literature, and a suspected or established diagnosis was reported in all individuals from the testing program and clinical trials. Clinical signs were reported for 1026 individuals with ≥ 2 P/LP variants and for 154 individuals who had ≥ 2 variants with ≥ 1 VUS (average of 3.6 and 3.9 clinical signs per patient, respectively). Among individuals with ≥ 2 P/LP variants for whom age and phenotypes were provided (*n* = 831), the frequency of cardiac phenotypes was highest in those under 1 year. Elevations of creatine kinase (a category that includes rhabdomyolysis), abnormal muscle function/strength, and muscle fatigability/pain were more frequent in those ≥ 1 year, while hypoglycemia and liver abnormalities were reported at less than threefold and less than fourfold higher frequency, respectively, in those under 1 year compared to those 1 year and older (Figure 5A). Individuals who have ≥ 2 variants with ≥ 1 VUS show similar patterns of phenotypes by age groups (Figure 5B).

Clinical signs were reported for 238 individuals with a single P/LP/VUS LC‐FAOD gene variant (heterozygous). When evaluated by age (*n* = 194), the frequencies of hypoglycemia and liver abnormalities were lower, and the frequencies of abnormal muscle function/strength, muscle fatigability/pain, and elevations of creatine kinase were higher among the ≥ 1‐year age groups compared to those under 1 year of age. Cardiomyopathy was reported at nearly equal frequency in the < 1‐year and 1–12‐year age groups (Figure 5C).

Although all 61 multiple‐heterozygous individuals had a positive NBS and/or clinical suspicion of LC‐FAOD that triggered genetic investigation, no specific clinical signs were reported for these individuals.

### 3.2. Spectrum of LC‐FAOD Gene Variants

#### 3.2.1. *CPT1A* (CPT I)

Variants in *CPT1A* represent 5% of recurrent (*n* = 329) and 9% of unique (*n* = 114) variants in the database as identified from the literature search (*n* = 83), the FAOD gene panel program (*n* = 26), clinical programs (*n* = 1), or multiple sources (*n* = 4). The 295 recurrent variants were found among 191 genotypes (89 unique), of which 29 had ≥ 2 P/LP variants and 31 had ≥ 2 variants with ≥ 1 VUS in *CPT1A* (remainder single‐ or multiple‐heterozygous genotypes). Classifications for the 114 unique variants are 22 P, 28 LP, 62 VUS, 1 benign, and 1 with unknown classification. Fifty‐eight *CPT1A* (50%) variants and 59 (53%) *CPT1A* genotypes were encountered only once in the database.

The most common *CPT1A* variants are listed in Table 3, along with associated geographies and gnomAD frequencies, and the most common *CPT1A* genotypes are listed in Table 4, along with geographies and clinical phenotypes. Hypoglycemia is the first or second most common clinical phenotype reported for each of the frequent variants and genotypes. The c.1436C > T (p.Pro479Leu) variant, which has been described as a common variant among native populations in the United States and Canada, is the second most frequent variant; homozygosity for this variant is the second most frequent *CPT1A* genotype in the database. While previous studies have reported this variant as having a mild phenotype [1, 8, 9, 25, 26], hypoglycemia, which can cause severe, life‐threatening outcomes, was reported in over half of individuals who had phenotypes reported and were homozygous for the p.Pro479Leu variant (Table 4).

**Table 3:** Six most common recurring *CPT1A* variants^a^.

Variant [classification] % of *CPT1A* variants	Times observed	Geographies	gnomAD frequency
c.100 T > C (p.Ser34Pro) [P] 14%	46	New Zealand, United States	Not found in gnomAD
c.1436C > T (p.Pro479Leu) [P] 11%	37	Canada, Greenland, Japan, United States	3.04e − 5
c.1364A > C (p.Lys455Thr) [LP] 5%	18	Finland	9.05e − 5
c.2129G > A (p.Gly710Glu) [LP] 5%	17	Canada, France	1.86e − 6
c.1318G > A (p.Ala440Thr) [VUS] 2%	7	China, Netherlands	1.24e − 6
c.823G > A (p.Ala275Thr) [benign] 2%	7	United States	6.45e − 2
c.740C > T (p.Pro247Leu) [VUS] 2%	7	China, Turkey	4.96e − 6

^a^Top 5 most frequent variants, including multiple with the same frequency.

**Table 4:** Six most common *CPT1A* genotypes^a^.

Genotype [variant classifications] % of *CPT1A* genotypes	Times observed/# reporting phenotypes	Geographies	Clinical phenotypes^a^ (*n*)
c.100 T > C (p.Ser34Pro)/c.100 T > C (p.Ser34Pro) [P/P] 12%	23/0	New Zealand, United States	N/A
c.1436C > T (p.Pro479Leu)/c.1436C > T (p.Pro479Leu) [P/P] 8%	16/8	Canada	Hypoglycemia (4), elevations of creatine kinase (1), hypoglycemic seizure (1), liver abnormality (1), loss of consciousness (1), impaired consciousness (1), abnormal muscle function/strength (1), seizure (1), vomiting (1)
c.2129G > A (p.Gly710Glu)/c.2129G > A (p.Gly710Glu) [LP/LP] 3%	6/6	Canada, France	Encephalopathy (5), hypoglycemia (5), diarrhea (4), liver abnormality (4), seizure (4)
c.1364A > C (p.Lys455Thr)/c.1364A > C (p.Lys455Thr) [LP/LP] 3%	5/4	Finland	Hypoglycemia (3), abnormality of metabolism/homeostasis (2), liver abnormality (2), anemia (1), hyperammonemia (1), hyperlipidemia (1), icterus (1), lethargy (1), loss of consciousness (1), mild disturbance of consciousness (1), nausea (1), thrombocytopenia (1), transient loss of consciousness (1), vomiting (1)
c.1318G > A (p.Ala440Thr)/c.1318G > A (p.Ala440Thr) [VUS/VUS] 3%	3/0	^b^	^b^
c.2122A > C (p.Ser708Arg)/c.2122A > C (p.Ser708Arg) [VUS/VUS] 3%	3/3	^b^	^b^

*Note:* Cardiac‐other: structural heart defects not expected to be related to disease. Muscle phenotypes were grouped into three categories: elevations of creatine kinase (e..g., rhabdomyolysis), muscle fatiguability/pain, and abnormal muscle function/strength.

^a^Top 5 most frequent genotypes and clinical phenotypes, including multiple with the same frequency.

^b^Demographics and clinical details are not reported for genotypes reported from fewer than five individuals.

The distribution of *CPT1A* variants by type and protein effect shows a similar pattern to that described for the overall database (Table 2). SNVs make up 89% (*n* = 101) of the 114 unique *CPT1A* variants, with most causing missense protein changes (*n* = 76/101, 75% of SNVs) (Tables 2). Twenty‐four (31%) of these missense variants are classified as P or LP.

The most common clinical phenotypes reported for individuals with ≥ 2 P/LP variants in *CPT1A* (*n* = 46 reporting) were hypoglycemia (67%), liver abnormality (46%), encephalopathy (24%), and diarrhea, seizure, and vomiting (all 22%) (Table 5). Thirty‐nine of these individuals had both age of diagnosis and phenotypes reported, including 15 patients aged < 1 year and 23 patients aged 1–12 years. Hypoglycemia (68%) and liver abnormalities (50%) were the two most common phenotypes in both of these age groups (Figure S4A).

**Table 5:** Clinical phenotypes (*n*) occurring in > 2 individuals with ≥ 2 P/LP variants for each disease.

CPT I (*n* = 46)	CACT (*n* = 58)	CPT II (*n* = 279)	LCHAD/TFP^a^ (*n* = 295)	TFP (*n* = 70)	VLCAD (*n* = 278)
Hypoglycemia (31)	Hypoglycemia (32)	Elevations of creatine kinase (196)	Elevations of creatine kinase (98)	Abnormal muscle	Elevations of creatine kinase (164)
Liver abnormality (21)	Liver abnormality (23)	Muscle fatiguability/pain (101)	Hypoglycemia (97)	Function/strength (37)	Muscle fatiguability/pain (98)
Encephalopathy (11)	Abnormal muscle function/strength (22)	Abnormal muscle function/strength (60)	Retinal/choroid disorder (86)	Elevations of creatine kinase (29)	Hypoglycemia (80)
Diarrhea (10)	Arrhythmia (20)	Renal insufficiency/failure (36)	Liver abnormality (85)	Peripheral neuropathy (26)	Abnormal muscle function/strength (73)
Seizure (10)	Hyperammonemia (20)	Liver abnormality (33)	Abnormal muscle function/strength (69)	Abnormality of metabolism/homeostasis (16)	Liver abnormality (47)
Vomiting (10)	Pulmonary/respiratory (17)	Pulmonary/respiratory (29)	Cardiomyopathy (63)	Cardiomyopathy (15)	Cardiomyopathy (45)
Abnormality of metabolism/homeostasis (9)	Cardiac arrest (16)	Hypoglycemia (26)	Peripheral neuropathy (58)	Hypoglycemia (14)	Abnormality of metabolism/homeostasis (31)
Abnormal muscle function/strength (6)	Abnormality of metabolism/homeostasis (14)	Arrhythmia (21)	Cardiac other (32)	Pulmonary/respiratory (11)	Lethargy (29)
Dehydration (6)	Lethargy (10)	Cardiac‐other (21)	Vomiting (30)	Liver abnormality (10)	Pulmonary/respiratory (24)
Developmental delay (5)	Cardiac‐other (9)	Abnormality of metabolism/homeostasis (17)	Abnormality of metabolism/homeostasis (29)	Cardiac‐other (8)	Vomiting (22)
Lethargy (5)	Cardiomyopathy (9)	Fever (17)	Muscle fatiguability/pain (25)	Muscle fatiguability/pain (8)	Cardiac‐other (21)
Hepatic encephalopathy (4)	Hypothermia (9)	Seizure (17)	Cardiac arrest (19)	Hypoparathyroidism (6)	Seizure (17)
Respiratory tract infection (4)	Seizure (9)	Abnormal renal morphology (16)	Decreased body weight (18)	Renal insufficiency/failure (5)	Arrhythmia (12)
Cardiac‐other (3)	Coma (5)	Lethargy (14)	Lethargy (16)	Developmental delay (4)	Hypothermia (9)
Gastrointestinal‐other (3)	Encephalopathy (4)	Cardiomyopathy (13)	Developmental delay (15)	Respiratory tract infection (4)	Coma (7)
Hyperammonemia (3)	Developmental delay (4)	Hyperammonemia (11)	Seizure (13)	Seizure (4)	Diarrhea (7)
	Gastrointestinal‐other (4)	Asthenia (9)	Absent Achilles reflex (11)	Annemia (3)	Fever (7)
	Cyanosis (3)	Vomiting (8)	Arrhythmia (8)	Difficulty running (3)	Poor feeding (7)
	Hypotension (3)	Cardiac arrest (6)	Diarrhea (8)	Fever (3)	Decreased body weight (6)
	Vomiting (3)	Gastrointestinal‐other (5)	Fever (8)	Hyperammonemia (3)	Dehydration (6)
		Periventricular calcifications (4)	Respiratory tract infection (8)	Lethargy (3)	Developmental delay (6)
		Encephalopathy (3)	Encephalopathy (7)	Retinal/choroid disorder (3)	Gastrointestinal‐other (6)
		Macrocephaly (3)	Jaundice (7)	Vomiting (3)	Renal insufficiency/failure (6)
		Micrognathia (3)	Gastrointestinal‐other (6)		Autism/behavior (5)
		Oligohydramnios (3)	Absent deep tendon reflexes (5)		Cardiac arrest (4)
		Sudden death (3)	Coma (5)		Diminished reflexes (4)
		Ventriculomegaly (3)	Ataxia (4)		Hypertrophy (4)
			Renal insufficiency/failure (4)		Pericardial effusion (4)
			HELLP syndrome (3)		Sudden death (4)
			Hyperammonemia (3)		Deceased (3)
			Hypotension (3)		Distress (3)
			Poor feeding (3)		Reye syndrome–like episodes (3)

*Note:* Cardiac‐other: structural heart defects not expected to be related to disease. Muscle phenotypes were grouped into three categories: elevations of creatine kinase (e.g., rhabdomyolysis), muscle fatiguability/pain, and abnormal muscle function/strength. See supporting information for a full description of phenotype curation and groupings.

Abbreviation: HELLP: hemolysis, elevated liver enzyme level, and low platelet level.

^a^Phenotypes associated with variants in *HADHA*, which may be associated with LCHAD or TFP.

#### 3.2.2. *SLC25A20* (CACT)

*SLC25A20* variants represent 4% of recurrent (*n* = 307) and 6% of unique (*n* = 74) variants in the database as identified from the literature search (*n* = 53), LC‐FAOD gene panel programs (*n* = 10), clinical programs (*n* = 1), or multiple sources (*n* = 10). The 307 recurrent variants were found among 187 genotypes (72 unique), of which 40 had ≥ 2 P/LP variants,12 had ≥ 1 VUS, and the remainder were single or multiple heterozygous for variants in *SLC25A20.* Classifications for the 74 unique variants were 37 P, 12 LP, 23 VUS, and 2 unknown. Thirty (40%) *SLC25A20* variants and 36 (38%) genotypes were encountered only once in the database.

The most common *SLC25A20* variants are listed in Table 6, along with associated geographies and gnomAD frequencies, and the most common *SLC25A20* genotypes are listed in Table 7, along with associated geographies and clinical phenotypes.

**Table 6:** Six most common recurring *SLC25A20* variants^a^.

Variant [classification] % of *SLC25A20* variants	Times observed	Geographies	gnomAD frequencies
c.199‐10 T > G (intronic) [LP] 33%	102	Australia, China, France, Japan, Malaysia, Saudi Arabia, Taiwan, United States	2.54e − 5
c.713A > G (p.Gln238Arg) [P] 7%	23	India, United States	Not found in gnomAD
c.804del (p.Phe269Serfs∗4) [P] 4%	13	China, France, Japan, United States	3.10e − 6
c.326+1del (intronic) [P] 4%	12	Australia, Brazil, Canada, United States	5.58e − 5
c.82G > T (p.Gly28Cys) [P] 2%	7	India, Norway, United Arab Emirates, United Kingdom	2.49e − 5
c.270del (p.Phe91Leufs∗38) [P] 2%	7	China	1.24e − 6

^a^Top 5 most frequent variants, including multiple with the same frequency.

**Table 7:** Six most common recurring *SLC25A20* genotypes^a^.

Genotype [variant classifications] % of *SLC25A20* genotypes	Times observed/# reporting phenotypes	Geographies	Clinical phenotypes^a^
c.199‐10 T > G (intronic)/c.199‐10 T > G (intronic) [LP/LP] 20%	37/18	Australia, China, France, Malaysia, Saudi Arabia, Taiwan, United States	Abnormal muscle function/strength (7), hypoglycemia (7), lethargy (6), arrhythmia (5), pulmonary/respiratory (5), cardiac arrest (2)
c.713A > G (p.Gln238Arg) [P] 11%	21/0	India	N/A
c.199‐10 T > G (intronic) [LP] 8%	15/2	China, France, United States	Abnormality of metabolism/homeostasis (1), cardiomyopathy (1), developmental delay (1), elevations of creatine kinase (1), failure to thrive (1), feeding difficulties (1), hypoglycemia (1), abnormal muscle function/strength (1), pulmonary/respiratory (1)
c.804del (p.Phe269Serfs∗4) [P] 3%	6/2	China, Japan, United States	Abnormality of metabolism/homeostasis (1), elevations of creatine kinase (1), hypertension (1), hypoglycemia (1), lack of alertness (1), abnormal muscle function/strength (1), paleness (1), poor reaction (1), pulmonary/respiratory (1), rapid breathing (1), spitting (1), swelling of the right heel (1)
c.824G > A (p.Arg275Gln)/c.824G > A (p.Arg275Gln) [LP/LP] 2%	3/3	^b^	^b^
c.82G > T (p.Gly28Cys)/c.82G > T (p.Gly28Cys) [P/P] 2%	3/2	^b^	^b^

*Note:* Cardiac‐other: structural heart defects not expected to be related to disease. Muscle phenotypes were grouped into three categories: elevations of creatine kinase (e.g., rhabdomyolysis), muscle fatiguability/pain, and abnormal muscle function/strength. N/A: no phenotypes reported.

^a^Top 5 most frequent genotypes and clinical phenotypes, including multiple with the same frequency.

^b^Geographies and clinical details are not reported for genotypes occurring in < 5 individuals.

The distributions of *SLC25A20* variants by type and protein effect show a similar pattern to that described for the overall database (Table 2). SNVs make up 76% (*n* = 56) of the 74 unique variants in this gene, with most causing missense protein changes (*n* = 35/56, 62% of SNVs). Nineteen (54%) of these missense variants are classified as P or LP.

The five most common clinical phenotypes reported among 58 individuals with ≥ 2 P/LP *SLC25A20* variants are hypoglycemia (55%), liver abnormality (40%), abnormal muscle function/strength (38%), arrhythmia (34%), and hyperammonemia (34%) (Table 5). Age of diagnosis and phenotypes were reported for 50 individuals with ≥ 2 P/LP *SLC25A20* variants; all but two of whom were in the < 1‐year age group; thus, comparisons between age groups were not feasible (Figure S4B).

#### 3.2.3. *CPT2* (CPT II)

Variants in *CPT2* comprise 21% of recurrent (*n* = 1484) and 17% of unique (*n* = 203) variants in the database as identified from the literature search (*n* = 146), LC‐FAOD gene panel programs (*n* = 26), clinical programs (*n* = 5), or multiple sources (*n* = 26). The 1484 recurrent variants were found among 908 genotypes (203 unique), of which 476 had ≥ 2 P/LP *CPT2* variants, 61 had ≥ *CPT2* variants with ≥ 1 VUS, and the remainder were single‐heterozygous or multiple‐heterozygous genotypes. Classifications for the 203 unique variants are 78 P, 41 LP, 77 VUS, 5 unknown, and 2 likely benign. One hundred and seven (53%) *CPT2* variants and 78 (26%) *CPT2* genotypes were encountered only once in the database.

The most common *CPT2* variants are listed in Table 8, along with associated geographies and gnomAD frequencies, and the most common *CPT2* genotypes are listed in Table 9, along with associated geographies and clinical phenotypes. The p.Ser113Leu P variant is most common (48%) and is present in the top four most common genotypes. Overall, the most common clinical phenotypes reported among the common genotypes are muscle‐related and consistent with a myopathic disease presentation (onset observed after age 1 year).

**Table 8:** Five most common recurring *CPT2* variants.

Variant [classification]% of *CPT2* variants	Times observed	Geographies	gnomAD frequency
c.338C > T (p.Ser113Leu) [P] 48%	713	Argentina, Australia, Austria, Bahrain, Belgium, Brazil, Canada, Chile, Denmark, France, Germany, Greece, Guatemala, Iran, Israel, Italy, Japan, Lebanon, Macedonia, Netherlands, New Zealand, Norway, Pittsburgh, Portugal, Spain, Taiwan, Turkey, United Kingdom, United States	1.67e − 3
c.1239_1240del (p.Lys414Thrfs∗7) [P] 4%	54	France, Germany, Israel, Italy, United States	1.19e − 4
c.1148 T > A (p.Phe383Tyr) [P] 4%	52	China, France, Japan, Korea, Taiwan	5.58e − 6
c.1891C > T (p.Arg631Cys) [P] 3%	49	Canada, China, Germany, Italy, Japan, Mexico, Netherlands, United States	2.04e − 5
c.149C > A (p.Pro50His) [P] 3%	46	Australia, Brazil, Canada, Denmark, France, Germany, Italy, Norway, Spain, United States	3.31e − 4

**Table 9:** Five most common recurring *CPT2* genotypes.

Genotype [variant classifications] % of *CPT2* genotypes	Times observed/# reporting phenotypes	Geographies	Clinical phenotypes^a^
c.338C > T (p.Ser113Leu)/c.338C > T (p.Ser113Leu) [P/P] 19%	168/104	Argentina, Austria, Belgium, Canada, Denmark, France, Germany, Greece, Iran, Israel, Italy, Japan, Macedonia, Netherlands, New Zealand, Portugal, Spain, Turkey, United Kingdom, United States	Elevations of creatine kinase (92), fatiguability (48), abnormal muscle function/strength (23), renal insufficiency/failure (12), fever (8)
c.338C > T (p.Ser113Leu)/c.1239_1240del (p.Lys414Thrfs∗7) [P/P] 2%	18/5	Germany, Italy, United States	Elevations of creatine kinase (5), muscle fatiguability/pain (2), amenorrhoeic (1), hypoglycemia (1), inability to move legs for several hours (1), muscle stiffness (1), renal insufficiency/failure (1)
c.338C > T (p.Ser113Leu)/c.1239_1240del (p.Lys414Thrfs∗7)|c.1342 T > C (p.Phe448Leu) [P/P] 1%	12/7	Germany, United States	Elevations of creatine kinase (7), muscle fatiguability/pain (4), abnormal muscle function/strength (2)
c.338C > T (p.Ser113Leu)/c.149C > A (p.Pro50His) [P/P] 1%	12/3	Brazil, Denmark, France, Germany, United States	Elevations of creatine kinase (2), muscle fatiguability/pain (2), renal insufficiency/failure (1)
c.1891C > T (p.Arg631Cys)/c.1891C > T (p.Arg631Cys) [P/P] 1%	10/8	Germany, Italy	Elevations of creatine kinase (6), muscle fatiguability/pain (5), abnormal muscle function/strength (3), renal insufficiency/failure (2), cardiac‐other (1), cardiomyopathy (1), coma (1), fever (1), hypoglycemia (1), lethargy (1), liver abnormality (1), pulmonary/respiratory (1), seizure (1), severe brain damage (1), sweating (1), vomiting (1)

*Note:* Cardiac‐other: structural heart defects not expected to be related to disease. Muscle phenotypes were grouped into three categories: elevations of creatine kinase (e.g., rhabdomyolysis), muscle fatiguability/pain, and abnormal muscle function/strength.

^a^Top 5 most frequent clinical phenotypes, including multiple phenotypes with the same frequency.

The distribution of *CPT2* variants by type and protein effect shows a similar pattern to that described for the overall database (Table 2). SNVs make up 72% (*n* = 147) of the 203 unique *CPT2* variants, with most causing missense protein changes (*n* = 122/147, 83% of SNVs). Forty‐six percent of these missense variants are classified as P or LP.

The five most common phenotypes reported among individuals with ≥ 2 P/LP variants in *CPT2* are elevations of creatine kinase (70%), muscle fatiguability/pain (36%), abnormal muscle function/strength (21%), renal insufficiency/failure (13%), and liver abnormality (12%) (*n* = 279 reporting phenotypes, Table 5). When evaluated by age group, muscle‐related phenotypes were more common among individuals 1 year and older, while liver abnormality, hypoglycemia, and cardiac phenotypes were most common in patients < 1 year (*n* = 233 with age and phenotype reported, Figure S4C).

#### 3.2.4. *ACADVL* (VLCAD)

*ACADVL* variants represent the largest proportion of recurrent (*n* = 2752, 40%) and unique (546, 45%) variants in the database as identified from the literature search (*n* = 283), LC‐FAOD gene panel programs (*n* = 122), clinical programs (*n* = 5), or multiple sources (*n* = 136). The 2752 recurrent variants were found among 1738 genotypes (604 unique), of which 327 had ≥ 2 P/LP *ACADVL* variants, 291 had ≥ 2 *ACADVL* variants with ≥ 1 VUS, and the remainder were single‐heterozygous or multiple‐heterozygous genotypes. Classifications for the 546 unique variants are 248 P, 116 LP, 171 VUS, 2 likely benign, 1 benign, and 2 with conflicting predicted classifications. Two hundred fifty‐eight (47%) *ACADVL* variants and 195 (23%) VLCAD genotypes were encountered only once in the database.

The most common *ACADVL* variants are listed in Table 10, along with associated geographies and gnomAD frequencies, and the most common genotypes are listed in Table 11, along with associated geographies and clinical phenotypes. The previously reported prevalent variant, c.848 T > C (p.Val283Ala), is the most common *ACADVL* variant in the database, and p.Val283Ala homozygosity is the most common genotype. This variant shows a broad geographical distribution, while the homozygous genotype was only reported from Europe and Australia; the associated phenotypes are consistent with the mild phenotype described in the literature [9, 41, 42]. However, hypoglycemia, a potentially life‐threatening event, was reported in 11 individuals with the p.Val283Ala variant (2 homozygous and 9 heterozygous) (Table 11 and data not shown).

**Table 10:** Five most common recurring *ACADVL* variants.

Variant [classification] % of *ACADVL* variants	Times observed	Geographies	gnomAD frequency
c.848 T > C (p.Val283Ala) [P] 16%	445	Argentina, Australia, Brazil, Canada, Denmark, France, Germany, Italy, Netherlands, Norway, Poland, Portugal, Slovenia, Spain, Sweden, United States	1.59e − 3
c.65C > A (p.Ser22∗) [P] 3%	82	China, Israel, Japan, Saudi Arabia	6.37e − 7
c.1349G > A (p.Arg450His) [P] 2%	63	Australia, Canada, China, Denmark, Iran, Italy, Japan, Korea, Pakistan, Saudi Arabia, Spain, Taiwan, Netherlands, United States	1.55e − 5
c.1322G > A (p.Gly441Asp) [P] 2%	58	Australia, Canada, Denmark, Germany, Iran, Italy, Netherlands, United States	2.54e − 5
c.779C > T (p.Thr260Met) [P] 2%	44	Canada, Denmark, France, Germany, Japan, Saudi Arabia, United States	2.66e − 5

**Table 11:** Six most common recurring *ACADVL* genotypes^a^.

Genotype [variant classifications] % of *ACADVL* genotypes	Times observed/# reporting phenotypes	Geographies	Clinical phenotypes^a^
c.848 T > C (p.Val283Ala)/c.848 T > C (p.Val283Ala) [P/P] 5%	82/13	Australia, Canada, Denmark, France, Germany, Italy, Netherlands, Norway, Poland, Spain, Sweden, United States	Elevations of creatine kinase (3), cardiac‐other (2), decreased body weight (2), muscle fatiguability/pain (2), hyperbilirubinemia (2), hypoglycemia (2)
c.65C > A (p.Ser22∗)/c.65C > A (p.Ser22∗) [P/P] 3%	39/3	Japan, Saudi Arabia	Elevations of creatine kinase (3), cardiomyopathy (1)
c.104del (p.Pro35Leufs∗26)/c.104del (p.Pro35Leufs∗26) [P/P] 1%	14/11	Netherlands, United States	Elevations of creatine kinase (10), muscle fatiguability/pain (7), hypoglycemia (5), cardiomyopathy (4), arrhythmia (2), cardiac‐other (2)
c.1500_1502del (p.Leu502del)/c.1500_1502del (p.Leu502del) [P/P] 1%	11/8	Australia, Brazil, Denmark, France, Italy, Portugal, United States	Elevations of creatine kinase (6), muscle fatiguability/pain (4), abnormality of metabolism/homeostasis (2), dizziness (1), mobilization difficulties (1), renal insufficiency/failure (1)
c.1349G > A (p.Arg450His)/c.1349G > A (p.Arg450His) [P/P] < 1%%	9/6	Canada, China, Japan, Korea, Saudi Arabia, United States	Cardiac‐other (2), cardiomyopathy (2), elevations of creatine kinase (2), hypoglycemia (1), lethargy (1), liver abnormality (1), muscle fatiguability/pain (1), abnormal muscle function/strength (1)
c.848 T > C (p.Val283Ala)/c.1376G > A (p.Arg459Gln) [P/P] < 1%	9/0	Germany, United States	N/A

*Note:* Cardiac‐other: structural heart defects not expected to be related to disease. Muscle phenotypes were grouped into three categories: elevations of creatine kinase (e.g., rhabdomyolysis), muscle fatiguability/pain, and abnormal muscle function/strength.

^a^Top 5 most frequent genotypes and clinical phenotypes, including multiple with the same frequency.

The distribution of *ACADVL* variants by type and protein effect shows a similar pattern to that described for the overall database (Table 2). SNVs make up 75% (*n* = 410) of the 546 unique *ACADVL* variants, with most causing missense protein changes (*n* = 321/410, 78% of SNVs) (Table 2). Sixty percent (*n* = 195) of these missense variants are classified as P or LP.

The five most common clinical phenotypes reported for individuals with ≥ 2 P/LP variants in *ACADVL* are elevations of creatine kinase (59%), muscle fatiguability/pain (36%), hypoglycemia (29%), abnormal muscle function/strength (26%), and liver abnormality (17%) (*n* = 278, Table 5). Among 245 individuals with ≥ 2 P/LP variants in *ACADVL* and age of diagnosis and phenotypes reported, hypoglycemia, liver abnormality, and cardiomyopathy were most common in the < 1 age group but were still reported in older patients. Elevations of creatine kinase occurred in all age groups at an increasing frequency with age (Figure S4D).

#### 3.2.5. *HADHA* (LCHAD/TFP)

*HADHA* variants make up 25% of recurrent variants (*n* = 1745) and 12% of unique variants (*n* = 150) in the database as identified from the literature search (*n* = 89), LC‐FAOD gene panel programs (*n* = 34), clinical programs (*n* = 5), or multiple sources (*n* = 22). The 1745 recurrent variants were found among 983 genotypes (139 unique), of which 52 have ≥ 2 P/LP variants, 30 have ≥ 2 variants with ≥ 1 VUS in *HADHA*, and the remainder were single‐heterozygous or multiple‐heterozygous genotypes. Classifications for the 150 unique variants are 68 P, 16 LP, 60 VUS, 5 unknown, and 1 benign. Eighty‐three (55%) *HADHA* variants and 67 (8%) genotypes were encountered only once in the database.

The most common *HADHA* variants are listed in Table 12, along with associated geographies and gnomAD frequencies, and the most common *HADHA* genotypes are listed in Table 13, along with associated geographies and clinical phenotypes. The common LCHAD variant, *HADHA c*.1528G (p.Glu510Gln), is the most common variant in the database, making up 19% of all LC‐FAOD variants and 75% of *HADHA* variants. This variant is also present in five of the six most common *HADHA* genotypes. Of particular note, 109 of 126 individuals (87%) with retinal/choroid disorder reported carrying one (*n* = 48) or two (*n* = 61) copies of the p.Glu510Gln variant.

**Table 12:** Five most common recurring *HADHA* variants.

Variant [classification] % of variants	Times observed	Geographies	gnomAD frequency
c.1528G > C (p.Glu510Gln) [P] 77%	1317	Argentina, Australia, Brazil, Canada, Czechia, Denmark, England, Estonia, Finland, France, Germany, Ireland, Italy, Netherlands, Norway, Poland, Spain, Sweden, Turkey, United Kingdom, United States	1.34e − 3
c.274_278del (p.Ser92Lysfs∗10) [P] 2%	27	Czechia, France, Germany, United States	3.04e − 5
c.703C > T (p.Arg235Trp) [P] 1%	22	China, Czechia, France, Germany, Lebanon, United States	2.48e − 6
c.180+3A > G (intronic) [LP] 1%	20	Norway, United States	4.50e − 5
c.1678C > T (p.Arg560∗) [P] 1%	18	France, Norway, Netherlands, United Kingdom, United States	2.06e − 5

**Table 13:** Six most common recurring *HADHA* genotypes^a^.

Genotype [variant classifications] % of genotypes	Times observed/# reporting phenotypes	Geographies	Clinical phenotypes^a^
c.1528G > C (p.Glu510Gln)/c.1528G > C (p.Glu510Gln) [P/P] 67%	463/178	Argentina, Australia, Austria, Brazil, Canada, Czechia, Denmark, Estonia, Finland, France, Germany, Ireland, Netherlands, Norway, Poland, Spain, Sweden, Turkey, United Kingdom, United States	Hypoglycemia (63), retinal/choroid disorder (61), liver abnormality (57), elevations of creatine kinase (49), cardiomyopathy (44)
c.1528G > C (p.Glu510Gln)/c.274_278del (p.Ser92Lysfs∗10) [P/P] 2%	18/11	Czechia, France, Germany, United States	Hypoglycemia (7), elevations of creatine kinase (6), liver abnormality (4), retinal/choroid disorder (3), cardiac‐other (2), peripheral neuropathy (2)
c.1528G > C (p.Glu510Gln)/c.1678C > T (p.Arg560∗) [P/P] 1%	11/10	Netherlands, United Kingdom, United States	Elevations of creatine kinase (4), hypoglycemia (4), retinal/choroid disorder (4), abnormality of metabolism/homeostasis (2), cardiac‐other (2), coma (2), fatiguability (2), irritability (2), liver abnormality (2), muscle function (2)
c.1528G > C (p.Glu510Gln)/c.180+3A > G (Intronic) [P/LP] 1%	10/3	United States	Elevations of creatine kinase (3), muscle fatiguability/pain (2), diminished ankle reflexes (1)
c.1528G > C (p.Glu510Gln)/c.479_482delinsAATA (p.Ile160_Gln763delinsLys)	6/5	United States	Elevations of creatine kinase (4), hypoglycemia (3), cardiac arrest (2), liver abnormality (2), peripheral neuropathy (2), retinal/choroid disorder (2)
c.1828C > G (p.Arg610Gly)/c.2281 T > G (p.Phe761Val) [LP/LP] 1%	6/4	Germany	Absent deep tendon reflexes (3), peripheral neuropathy (3), ataxic gait (2), abnormal muscle function/strength (1), stumble (1)

*Note:* Cardiac‐other: structural heart defects not expected to be related to disease. Muscle phenotypes were grouped into three categories: elevations of creatine kinase (e.g., rhabdomyolysis), muscle fatiguability/pain, and abnormal muscle function/strength.

^a^Top 5 most frequent genotypes and clinical phenotypes, including multiple with the same frequency.

The distribution of *HADHA* variants by type and protein effect shows a similar pattern to that described for the overall database (Table 2). SNVs make up 72% (*n* = 108/150) of the unique *HADHA* variants, with most causing missense protein changes (*n* = 66/108, 61% of SNVs). Fifteen percent (*n* = 16) of these missense variants are classified as P or LP.

The most frequently reported clinical phenotypes among 295 individuals with ≥ 2 P/LP *HADHA* variants and phenotypes reported are elevations of creatine kinase (33%), hypoglycemia (33%), retinal/choroid disorder (29%), liver abnormality (29%), and abnormal muscle function/strength (23%) (Table 5).When evaluated by age group, hypoglycemia, liver abnormality, and cardiac phenotypes were more common among patients < 1 year than those 1 year or older, while muscle‐related phenotypes were more common among patients 1–12 years (*n* = 211 patients with age and phenotypes reported) (Figure S4E).

#### 3.2.6. *HADHB* (TFP)

Variants in *HADHB* make up 5% of recurrent (*n* = 330) and 11% of unique (*n* = 130) variants in the database as identified from the literature search (*n* = 92), LC‐FAOD gene panel programs (*n* = 30), clinical programs (*n* = 2), or multiple sources (*n* = 6). The 330 recurrent variants were found among 212 genotypes (119 unique), of which 66 have ≥ 2 P/LP variants and 32 have ≥ 2 variants with ≥ 1 VUS in *HADHB*; the remaining *HADHB* genotypes were single or multiple heterozygous. Classifications for the 130 unique variants are 46 P, 23 LP, 58 VUS, and 3 unknown. Sixty (46%) *HADHB* variants and 73 (46%) genotypes were reported only once in the database.

The most common *HADHB* variants are listed in Table 14, along with associated geographies and clinical phenotypes. The most common *HADHB* genotypes in individuals with ≥ 2 P/LP/VUS variants are listed in Table 15; associated geographies and phenotypes are not provided as these genotypes were reported in < 5 individuals.

**Table 14:** Eight most common recurring *HADHB* variants^a^.

Variant [classification]% *HADHB* of variants	Times observed	Geographies	gnomAD frequency
c.1331G > A (p.Arg444Lys) [LP] 4%	11	Japan	Not found in gnomAD
c.1175C > T (p.Ala392Val) [LP] 3%	8	Japan	1.88e − 6
c.739C > T (p.Arg247Cys) [LP] 3%	8	China, Germany, Japan, Korea	1.05e − 5
c.1165A > G (p.Asn389Asp) [P] 3%	7	China, France, Korea, United States	3.15e − 6
c.181C > T (p.Arg61Cys) [LP] 2%	7	Netherlands, United States	1.43e − 5
c.209+1G > C (Intronic) [P] 3%	7	France, Norway, Netherlands	6.47e − 7
c.397A > G (p.Thr133Ala) [VUS] 3%	7	Netherlands, United States	1.89e − 4
c.901G > A (p.Gly301Ser) [VUS] 3%	7	Brazil, Italy, United States	3.72e − 6

^a^Top 5 most frequent variants, including multiple variants with the same frequency.

**Table 15:** Three most frequently recurring *HADHB* genotypes with ≥ 2 P/LP/VUS.

Genotype [classification] % *HADHB* genotypes	Times observed/# reporting phenotypes	Geographies	Phenotypes
c.1175C > T (p.Ala392Val)/c.1175C > T (p.Ala392Val) [LP/LP] 1%	3/3	^a^	^a^
c.580C > T (p.Gln194∗) c.1115A > T (p.Asp372Val) [P/LP] 1%	3/2	^a^	^a^
c.693del (p.Ala232Leufs∗20)/c.881C > G (p.Pro294Arg) [P/LP] 1%	3/2	^a^	^a^

Note: The three most frequent genotypes with ≥ 2 P/LP/VUS *HADHB* genotypes were reported in three individuals each. Approx. 20 different ≥ 2 P/LP/VUS *HADHB* genotypes were reported in two individuals each and are not summarized here.

^a^Geographies and phenotypes are not reported for genotypes occurring in < 5 individuals.

The distribution of *HADHB* variants by type and protein effect shows a similar pattern to that described for the overall database (Table 2). SNVs make up 82% (*n* = 107) of the 130 unique variants, with most causing missense protein changes (*n* = 81/107, 76% of SNVs). Forty‐three percent of these missense variants are classified as P or LP.

The Top 5 clinical phenotypes reported among 70 individuals with > 2 P/LP *HADHB* variants and phenotypes reported are abnormal muscle function/strength (53%), elevations of creatine kinase (41%), peripheral neuropathy (37%), abnormality of metabolism/homeostasis (23%), and cardiomyopathy (21%) (Table 5). When evaluated by age of diagnosis, muscle‐related phenotypes and peripheral neuropathy were more common in patients 1–12 years, while abnormality of metabolism/homeostasis, hypoglycemia, cardiac phenotypes, pulmonary/respiratory phenotypes, and liver abnormality were most common in patients < 1 year (*n* = 53, Figure S4F).

### 3.3. Biochemical Findings of LC‐FAOD

**Figure 6:**
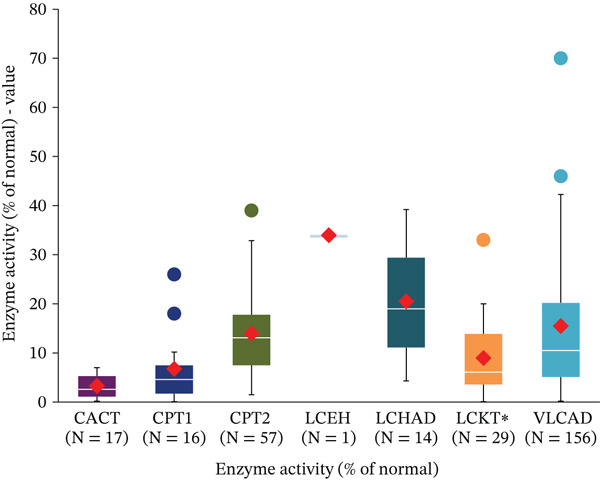
Enzyme activity (% of normal) for 290 individuals with ≥ 2 P/LP variants in an LC‐FAOD gene.  ^∗^LCKT: LKAT or LCTH. The number below each boxplot indicates the number of individuals with an enzyme activity result included in the plot. Red diamonds and horizontal pastel lines indicate median and mean values, respectively. For *HADHA* and *HADHB* genotypes impacting the TFP complex, the lowest deficient enzyme in the complex is represented, given that other enzyme activities in the complex may be differentially expressed. For non‐TFP genotypes where the impacted enzyme activity was not reported, other reported enzyme activities are not shown. Individuals with enzyme activities over 40% of normal are addressed further as follows: An individual with 70% VLCAD activity was diagnosed at age 14 years with an episodic, myopathic form of VLCAD and was shown to be a compound heterozygote with two temperature‐sensitive pathogenic *ACADVL* variants (p.Ala416Thr [P]/p.Arg450His [LP]) [49]. A 2‐year‐old with 46% VLCAD enzyme activity presented for sequencing following abnormal NBS. The individual′s sibling (2 years) was also tested and had 40% VLCAD enzyme activity. The siblings had no complaints and normal cardiac and neurological examinations but were found to be heterozygous for one pathogenic and one likely pathogenic missense variant (p.Gly441Asp [P]/p.Arg615Gln [LP] [50]. An individual with 42% VLCAD enzyme activity (age not reported) had abnormal acylcarnitine values, which triggered sequencing; no other clinical phenotypes were reported. Sequencing revealed compound heterozygosity for pathogenic and likely‐pathogenic missense variants in *ACADVL* (p.Ala416Thr [P]/p.Cys607Ser [LP]) [51].

Results from 485 enzyme activity assays, performed in lymphocytes or fibroblasts, were available; more than 60% of these test results were provided without associated normal values for the testing laboratory and could not be included in a combined analysis. Enzyme activity levels as a percentage of normal are plotted in Figure 6 for 290 patients with ≥ 2 P/LP LC‐FAOD gene variants, showing a wide window of residual enzyme activity levels in some LC‐FAOD types (Figure 6, Table 16). Additional clinical details, extracted from published literature, are provided for the three patients with a reported enzyme activity of 40% or greater (Figure 6).

**Table 16:** Enzyme activity ranges for LC‐FAOD P/LP genotypes, VUS‐containing genotypes, and single‐variant genotypes^a^.

LC‐FAOD type	# of individuals reporting for each enzyme	≥ 2 P/LP variants (*n* = 290)	≥ 2 variants, ≥ 1 VUS (*n* = 32)	Single P/LP/VUS variant (*n* = 111)
VLCAD (*ACADVL)*	VLCAD *n* = 236	Mean 11.4% of normal (range 0%–70.0%)	Mean 20.7% of normal (range 1.0%–54.1%)	Mean 35.4% of normal (range 5.0%–75.0%)
CPT I (*CPT1A*)	CPT I *n* = 18	Mean 5.9% of normal (range 0%–26.0%)	^a^	^a^
CPT II (*CPT2*)	CPT II *n* = 95	Mean 12.8% of normal (range 1.2%–39.0%)	^a^	Mean 29.1% of normal (range 4.0%–80.0%)
LCHAD/TFP (*HADHA*)	LCHAD *n* = 18, LCKT *n* = 13	Mean LCHAD 22.2% of normal (range 4.0%–39.0%), mean LCKT 8.2% of normal (range 0.0%–33.0%)	^a^	^a^
TFP (*HADHB*)	LCHAD *n* = 6, LCEH *n* = 1, LCKT *n* = 28	Mean LCHAD^a^, mean LCEH^a^, mean LCKT 11.1% of normal (range 2.3%–33.0%)	^a^	^a^
CACT (*SLC25A20*)	CACT *n* = 17	Mean 1.8% of normal (range 0%–6.8%)	^a^	^a^

^a^Results are not reported for tests/groups with data from < 5 individuals.

Similar results were reported for single heterozygous individuals (*n* = 111) and those with two variants and ≥ 1 VUS (*n* = 32) (Table 16). In general, genotypes with two P variants were associated with a low level of enzyme activity, while genotypes with ≤ 1 P/LP variant were associated with 50% of normal enzyme activity. However, there are noteworthy exceptions in both groups, as apparent by the mean enzyme activity values of < 40% for patients with one or more VUS or only a single LC‐FAOD gene variant and by the enzyme activity levels at or above the level expected for healthy carriers in some individuals with ≥ 2 P/LP variants (Table 16, Figure 6).

NBS blood spot acylcarnitine values were reported for 672 individuals with ≥ 2 P/LP LC‐FAOD gene variants and for 156 individuals with ≥ 2 variants and ≥ 1 VUS. Metabolite elevations were as expected, including some values as low as 0; further analyses are limited due to the heterogeneity of tests and specimen types (Figure S5A,B). Fasting plasma acylcarnitine values as reported among 32 patients with ≥ 2 P/LP/VUS variants were similar to NBS results.

### 3.4. Severe Outcomes Reported in LC‐FAOD

The most severe outcomes reported in LC‐FAOD include retinopathy, cardiac manifestations, and death. As described previously, retinal/choroid defects were reported for 126 individuals in the database, most of whom (*n* = 109) carried one or two copies of the common LCHAD variant, *HADHA* p.Glu510Gln. Cardiac manifestations (cardiac arrest, cardiomyopathy, increased cardiothoracic ratio, pericardial effusion, and arrhythmia) were reported for 252 individuals with ≥ 2 P/LP/VUS in an LC‐FAOD gene (*HADHA*, *n* = 83 [33%]; *ACADVL*, *n* = 68 [27%]; *CPT2*, *n* = 36 [14%]; *SLC25A20*, *n* = 35 [14%]; *HADHB*, *n* = 27 [11%], and *CPT1A*, *n* = 3 [1%]), 175 (75%) of whom were < 1 year at diagnosis (235/252 with age available) (Figure S6). One hundred seventy‐seven individuals with ≥ 2 P/LP/VUS in an LC‐FAOD gene were reported deceased (*HADHA*, *n* = 55 [31%]; *ACADVL*, *n* = 34 [19%]; *SLC25A20*, *n* = 36 [20%]; *CPT2*, *n* = 33 [19%]; *HADHB*, *n* = 18 [10%]; and *CPT1A*, *n* = 1 [1%]) with age of death ranging from 0 to 30 years. The most common variants among individuals with outcomes categorized as severe were *HADHA*:c.1528G > C (p.Glu510Gln) and *SLC25A20*:c.199‐10 T > G (intronic and splice region), and the most common genotype was homozygous *HADHA*:c.1528G > C (p.Glu510Gln) (*n* = 45, 19, and 33, respectively).

### 3.5. The LC‐FAOD Gene Database Website

**Figure 7:**
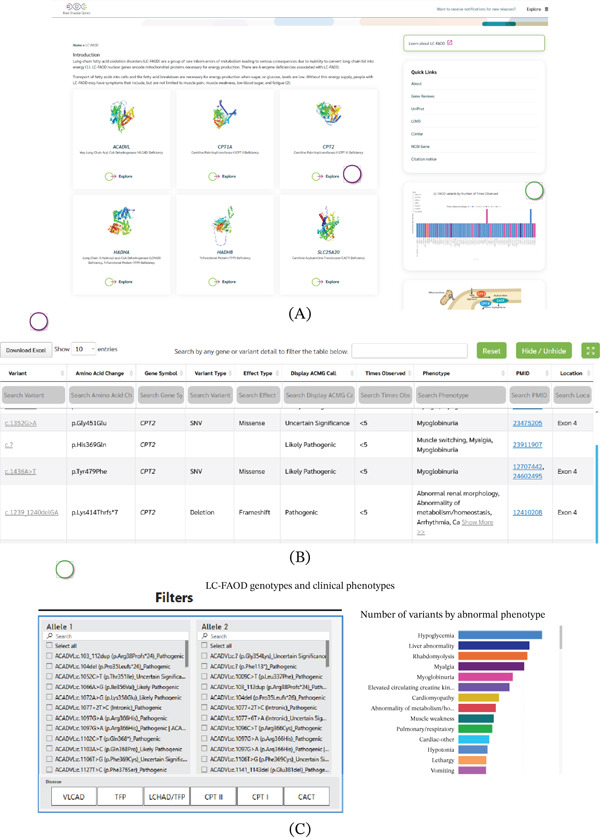
LC‐FAOD gene variant database website (accessed November 2025). (A) The rarediseasegenes.com landing page for LC‐FAOD. (B) From the LC‐FAOD landing page, users can navigate to a detailed variant table for each gene by clicking “Explore” under the gene name/protein structure, as illustrated here for *CPT2*. (C) Interactive summary analyses show genotype–phenotype and biochemical associations as well as aggregate variant metrics, as illustrated here for LC‐FAOD genotypes and phenotypes.

The LC‐FAOD database is a searchable, interactive online database, openly available to the global scientific and LC‐FAOD communities through a public website, https://www.rarediseasegenes.com/lc-faod/. The database features a variant‐level table for each of the six genes associated with LC‐FAOD, a genotype–phenotype table, interactive summary analyses, and a variant submission process to contribute variant data (Figure 7A–C).

The variant table provides each variant reported in HGVS nomenclature, predicted impact, reported and/or predicted variant classification, times observed, associated phenotypes, and publications reported with the variant. The variant table is searchable for custom queries, interactive to reflect the variant position relative to the gene structure, and dynamic to filter for variants located in a selected gene region of interest (Figure 7B).

The genotype–phenotype table displays biallelic genotypes with times observed, associated phenotypes, and publications reported with the genotype. This table is searchable for custom queries, and each genotype dynamically links to a genotypedetails page. Summary analyses show the aggregate variant metrics, geographic location, and interactive graphs of variant–phenotype and genotype–phenotype associations (Figure 7C). Genotypes are interactively displayed with associated enzyme activity and results of NBS. Care is taken to remove any possibility of identifiability when displaying LC‐FAOD genotype data. For example, identifying details such as phenotypes and geographical location are only displayed in aggregate for variants/genotypes found in at least five individuals in the database.

An online variant submission process enables users to contribute deidentified variants and associated data (Figure 7A). Users submit data through a webform and an Excel template that collects variant details in a standardized format, with patient consent previously collected. Once submitted, dedicated database personnel curate the variant data, with review by external medical and scientific experts prior to inclusion in the aggregate database.

## 4. Discussion

The LC‐FAOD gene database combines variants and associated data from a comprehensive literature review and LC‐FAOD genetic testing programs and represents the first centralized database for variants in all six LC‐FAOD genes. One thousand forty‐one unique P, LP, or VUS variants are reported from 4188 individuals. Missense variants represent the most common type of variant in the database (60%), but some variants that are more challenging to detect with NGS, such as subgenic CNVs and intronic variants, are also represented. The database only includes one variant in 3 ^′^ or 5 ^′^ untranslated regions and does not include any large structural variants that do not have breakpoints within the coding regions. The database is consistent with current understanding of LC‐FAOD variants in terms of relative prevalence, phenotypes, and landscape of variants.

The identification of individuals with abnormal acylcarnitine profiles, enzyme activity, and/or clinical presentation of disease who lack a clear genetic diagnosis (e.g., heterozygous or multiple heterozygous or those with one or more VUSs) suggests there might be undetected variants in these individuals, insufficient data for variant classification, or other mechanisms of disease manifestation. It has been demonstrated that up to one in seven P variants can be difficult to detect by NGS‐based detection methods, as NGS methods miss most intronic regions and do not always detect structural variants and CNVs effectively [52]. Thus, the lack of a genetic diagnosis does not rule out the presence of disease.

Consistent with previous findings, 49% of all variants and 71% of all genotypes were reported only once in the database. This diverse composition of gene variants among LC‐FAOD patients has a significant impact on the ability to predict pathogenicity and disease course. The three most common variants are *HADHA* c.1528G > C (p.Glu510Gln) (*n* = 1317), *CPT2* c.338C > T (p.Ser113Leu) (*n* = 713), and *ACADVL* c.848 T > C (p.Val283Ala) (*n* = 369). Clinical phenotypes reported among patients with these variants are consistent with previous reports, with the exception of *ACADVL* p.Val283Ala. Previous reports suggest that *ACADVL* p.Val283Ala causes a mild phenotype [9, 41, 42], but the database reports phenotypes associated with poor outcomes (e.g., hypoglycemia) in a small number of patients who are homozygous for this variant. While this observation is based on data from only two patients (of 13 reporting), it suggests that the variant is not always restricted to mild disease. Prior studies have also shown a lack of correlation between LC‐FAOD genotype and clinical phenotype, even among members of the same family. Data from more patients is needed to explore whether such correlations exist for some variants/genotypes. The LC‐FAOD gene variant database provides the opportunity to report data from large numbers of patients in a single repository where results can be compared and analyzed in aggregate.

Phenotype data highlight the similarities and differences in phenotypic expression of disease by type and by age. Hypoglycemia and muscular abnormalities (elevated CK, abnormal function/strength, or weakness/pain) were among the most frequently reported phenotypes among all LC‐FAOD types. LC‐FAOD phenotypes were also reported among single heterozygous individuals and can help establish a diagnosis in those who lack a definitive genetic diagnosis. The relatively low incidence of cardiac manifestations in individuals with ≥ 2 P/LP *SLC25A20* variants is surprising, as cardiac manifestations appear nearly ubiquitously in individuals with CACT in the existing clinical literature [15–17].

Enzyme activity levels showed a wide window of residual activity among patients with ≥ 2 P/LP variants in the LC‐FAOD gene. Most variants that have been reported in the literature as having low enzyme activity were associated with a low percentage of normal enzyme activity in this database analysis. When enzyme activity is higher than expected in the face of clinical symptoms, other methods should be used to assess the diagnosis. The data contained herein highlight the need for future publications of LC‐FAOD genotypes to include supporting biochemical and phenotype data to further understand the impact of gene variants on disease.

The reports of false‐negative NBS results, particularly for CPT II, highlight that some individuals with LC‐FAOD can be missed through NBS. Additionally, some individuals with a single‐heterozygous *ACADVL* variant may have MS/MS analyte levels that overlap into the VLCAD‐affected range, as well as GA‐II patients, including for five MS/MS analytes (C12, C14:1, C12:1, C14, and C14:2) and two analyte ratios (C14:1/C2 and C14:1/C16) [53]. Importantly, all six diseases are not consistently included in NBS programs around the globe, and the diagnoses should always be considered in the appropriate clinical context, especially in individuals born in countries where LC‐FAODs are not screened at birth. The proportion of LC‐FAOD types observed in the database overall differs compared to those observed in association with abnormal NBS, suggesting that some types might be underdiagnosed by NBS. These results emphasize that suspicion of LC‐FAOD, based on clinical presentation or a positive NBS result, must be confirmed with follow‐up biochemical and molecular genetic testing. Performing multiple tests helps overcome the limitations of detection for each test to provide an accurate diagnosis for the patient and avoid false negatives.

## 5. Limitations

The analyses described herein are limited by the retrospective nature of the data collection. We were not able to determine whether patients with clinical and biochemical data provided in case reports or genetic testing forms were on fat‐restricted diets and/or receiving treatment for LC‐FAOD. The analyses are also contingent on the variant classifications as interpreted at the time of analysis; these interpretations are not static and often change over time when new data become available. Due to the high degree of genetic heterogeneity and the limited number of patients with clinical phenotypes reported, it was not possible to determine if genotype–phenotype correlations exist, except for the few common variants in each gene. The enzyme functional results must also be interpreted with caution, as the number of patients with evaluable data was low, and the analysis shows values from different labs and different cell types (lymphocyte vs. fibroblasts) on the same plot. Further, and not discussed in this manuscript, data were mostly representative of North American and European countries and were lacking from South America, Asia, and Africa, highlighting the need for global publications on variant–phenotype data.

## 6. LC‐FAOD Database Curation and Updates

The LC‐FAOD database is overseen and curated by a qualified scientific committee including clinical, metabolic, and molecular genetic experts. New LC‐FAOD gene variants and/or new patient‐associated data, originating from online submissions or genetic testing labs, will be added regularly. Following review by the scientific committee, all data displays and analysis pages will be updated, and new variants will be shared twice‐yearly with ClinVar and the Leiden Open Variation Database.

## 7. Conclusions

Gene locus–specific databases are critically important in rare diseases, where clinical information associated with gene variants is scarce, and VUSs are frequent and insufficient to support a molecular diagnosis. This comprehensive database of LC‐FAOD disease gene variants increases the number of reported unique variants by 328 and provides additional classifications for 126 unique variants, compared to P/LP/VUS variants present in ClinVar prior to contributions from the FAOD testing program. The database is freely available and globally accessible and will be a valuable resource for both researchers and clinicians looking to better understand the implications of genetic testing results and to access data relevant to interpreting findings from NBS.

## Author Contributions

Nicole Miller and Vanessa Rangel Miller developed the concept for the LC‐FAOD database and led data collection, curation, and website development. Heather Richbourg, Omid Khazaie Japalaghi, and Sean Daugherty compiled and curated the database, performed summary analyses, and developed the website. Vanessa Rangel Miller, Heather Richbourg, and Omid Khazaie Japalaghi developed the outline and first draft. All authors contributed to data interpretation and reviewed the manuscript and database. Heather Richbourg, Vanessa Rangel Miller, and Omid Khazaie Japalaghi contributed equally to the work.

## Funding

This study was funded by Ultragenyx Pharmaceutical, 10.13039/100013220.

## Disclosure

All authors approved the final version of the manuscript.

## Ethics Statement

The authors have nothing to report.

## Consent

All patients who provided samples through the genetic testing program consented to have their deidentified data published.

## Conflicts of Interest

J. Vockley received research funding from Ultragenyx Pharmaceutical Inc. L. Korngut received ad board honoraria and research funding from Ultragenyx Pharmaceutical Inc. P. Baker II speaks in a lecture series, received an educational grant from Ultragenyx Pharmaceutical Inc., and is an investigator on a study sponsored by Ultragenyx Pharmaceutical Inc. A. Khan received research funding and consulting fees from Ultragenyx Pharmaceutical Inc. M. AlSayed is an investigator on a study sponsored by Ultragenyx Pharmaceutical Inc. H. Richbourg, O. K. Japalaghi, V. Rangel Miller, and S. Daugherty are employees and shareholders of Ultragenyx Pharmaceutical Inc. N. Miller is a shareholder and former employee of Ultragenyx Pharmaceutical Inc. M. Kiel is an employee and shareholder of Genomenon. S. Monteleone is an employee of Genomenon. T. Ekstein is an employee of Labcorp (formerly Invitae).

## Supporting Information

Additional supporting information can be found online in the Supporting Information section.

## Supporting information


**Supporting information 1** Figure S1: Geographic location for 2350 individuals with ≥ 2 P/LP/VUS LC‐FAOD gene variants and geography reported∗. Figure S2: Frequency of LC‐FAOD subtype by age category for 1327 of 2372 (56%) individuals with ≥ 2 LP/P variants and age of diagnosis recorded. Figure S3: Abnormal newborn screening (NBS) results were reported for 1370 individuals (of 1399 reporting). Figure S4: Phenotypes by age group among individuals with ≥ 2 P/LP variants in an LC‐FAOD gene. Figure S5: NBS blood spot acylcarnitine values as reported for (a) 241 patients with ≥ 2 P/LP variants and (b) 48 individuals with 2 variants, 1 or more VUSs. Figure S6: Cardiac manifestations occurred in 252 individuals with ≥ 2 P/LP/VUS.

**Supporting Information 2** Table S1: LC‐FAOD gene variants. List of variants and associated data for *ACADVL*, *CPT1A*, *CPT2*, *HADHA*, *HADHB*, and *SLC25A20*.

**Supporting information 3** Table S2: LC‐FAOD genotypes. List of genotypes and associated data for individuals with at least 2 P, LP, or VUS variants in *ACADVL*, *CPT1A*, *CPT2*, *HADHA*, *HADHB*, or *SLC25A20.*

**Supporting information 4** Table S3: Single‐heterozygous genotypes. List of genotypes and associated data for individuals with a single P, LP, or VUS variant in *ACADVL*, *CPT1A*, *CPT2*, *HADHA*, *HADHB*, or *SLC25A20.*

**Supporting information 5** Table S4: Double‐heterozygous genotypes. List of genotypes and associated data for individuals with P/LP/VUS variants in two different LC‐FAOD genes (*ACADVL*, *CPT1A*, *CPT2*, *HADHA*, *HADHB*, or *SLC25A20*).

**Supporting information 6** Table S5: LC‐FAOD gene variants unique to genetic testing programs. List of variants (and associated data) that were only identified in LC‐FAOD genetic testing programs.

## Data Availability

All LC‐FAOD variants and genotypes are available in the supporting information (Excel files). The LC‐FAOD database reports additional data in aggregate for each variant and genotype; it is available at http://www.rarediseasegenes.com/lcfaod. All novel LC‐FAOD gene variants reported here for the first time are listed in Table S4 and will be submitted to ClinVar (https://www.ncbi.nlm.nih.gov/clinvar/).
