# Supplementary material for: Long‐Chain Fatty Acid Oxidation Disorder Genes: A Comprehensive Genetic Database of LC‐FAOD Variants, Genotypes, and Phenotypes
Source: Hum Mutat. 2026 Jul 7;2026:6864813. doi: 10.1155/humu/6864813 (PMC13342283; doi:10.1155/humu/6864813)

Figure S1. Geographic location for 2350 individuals with ≥2 P/LP/VUS LC-FAOD gene variants and geography reported*

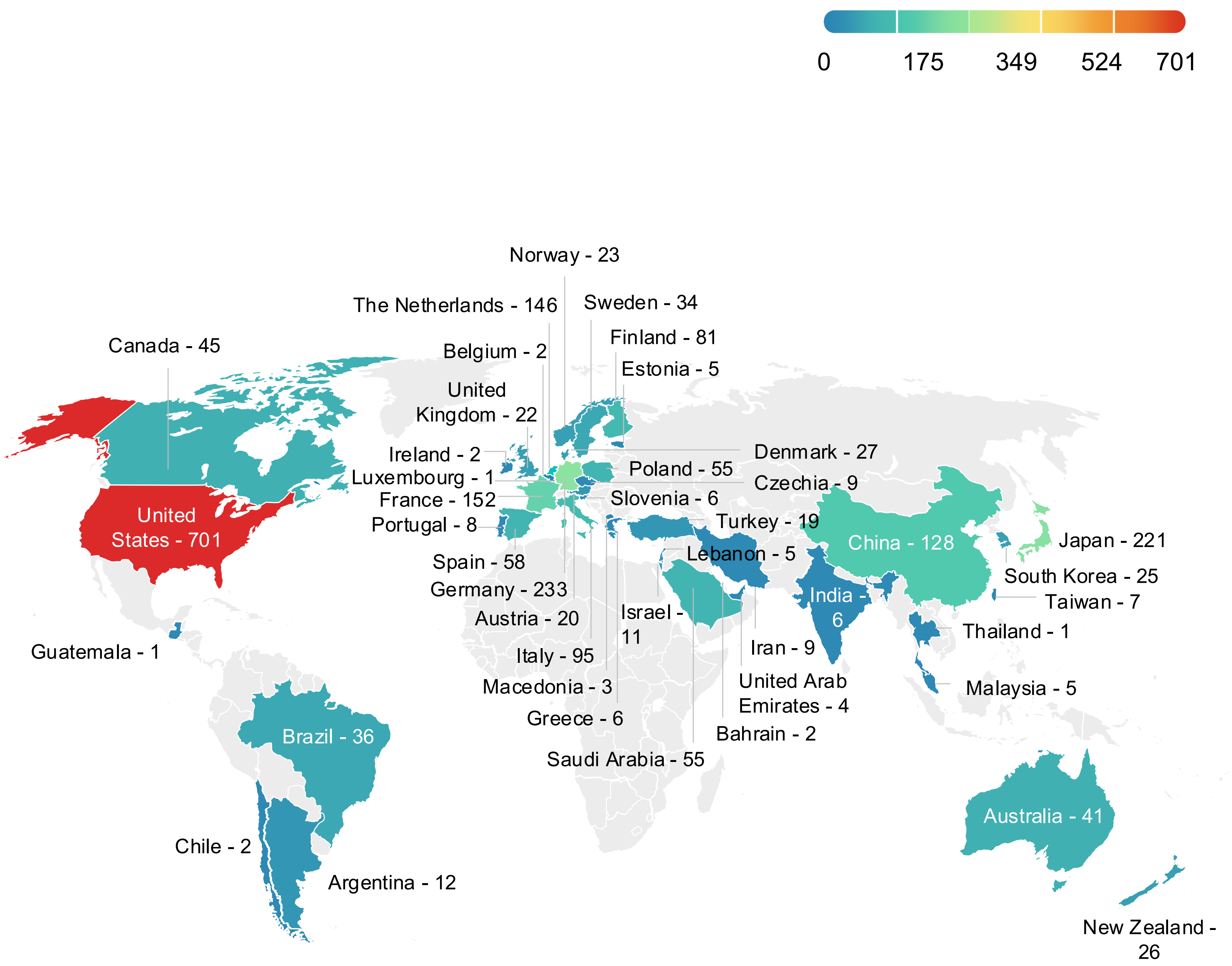

Figure S2. Frequency of LC-FAOD subtype by age category for 1327 of 2372 (56%) individuals with ≥2 LP/P variants and age of diagnosis recorded.

NBS for LC-FAOD began in the ~mid 2000's, and screening for all 6 LC-FAOD is not consistently available around the globe.

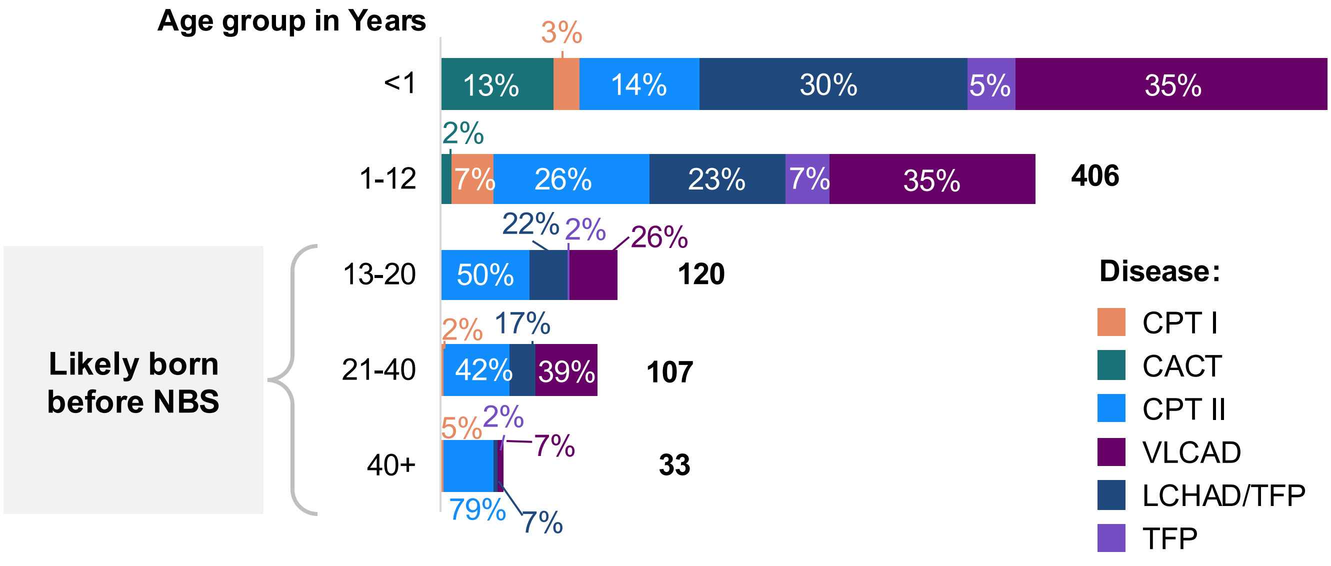

Figure S3. Abnormal newborn screening (NBS) results were reported for 1370 individuals (of 1399 reporting)

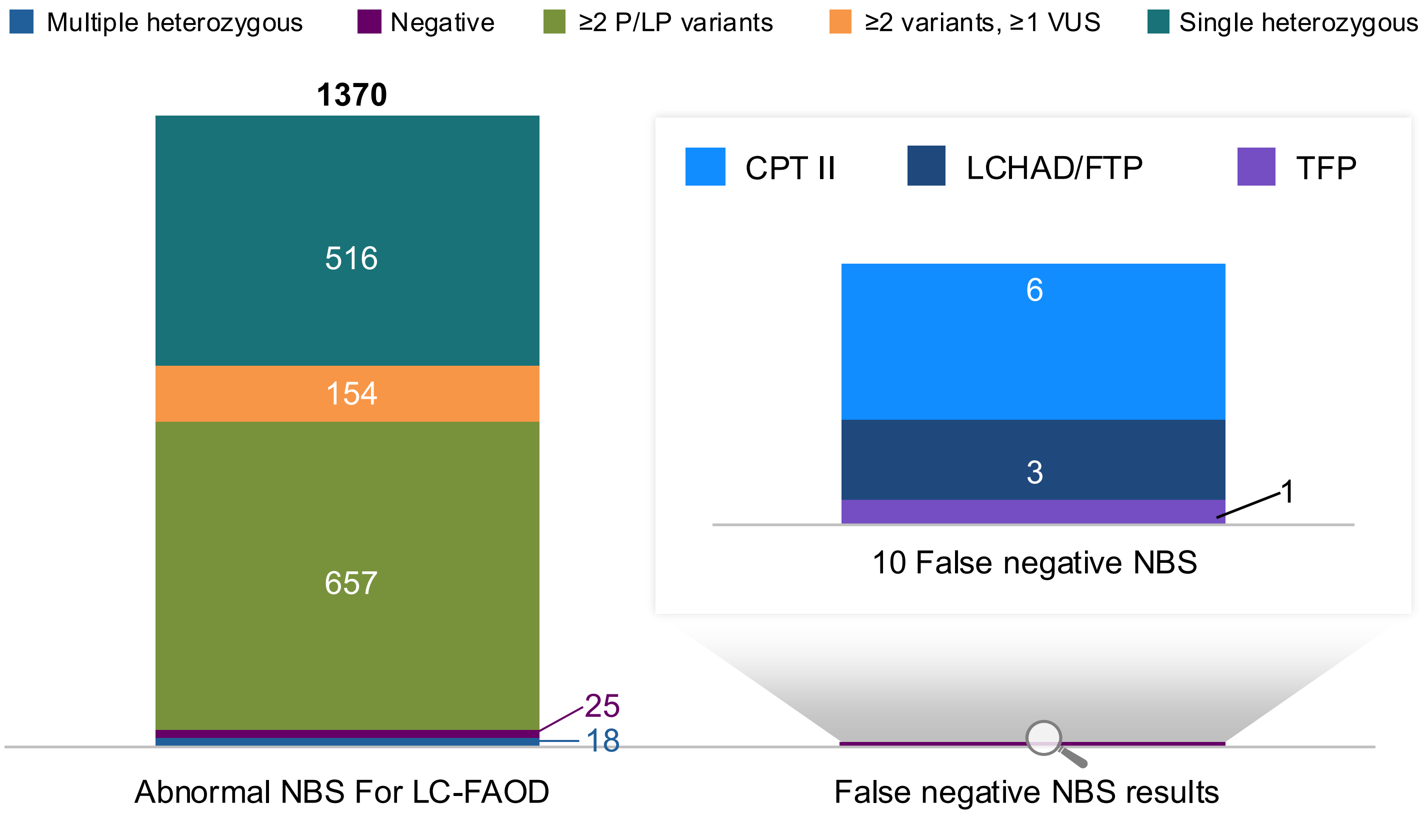

Figure S4. Phenotypes by age group among individuals with ≥2 P/LP variants in an LC-FAOD gene

Age Group (years): <1, 1-12, 13-20, 21-40, 40+

A. Phenotypes[a] by age group among individuals with ≥2 P/LP *CPT1A* variants

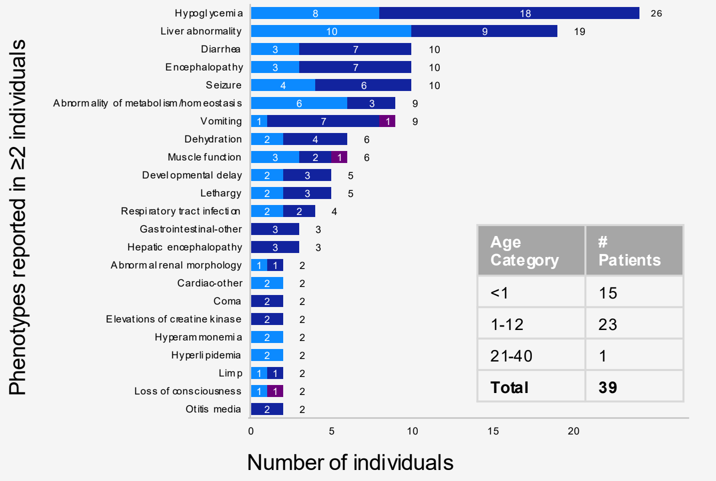

Age Category	# Patients
<1	15
1-12	23
21-40	1
**Total**	**39**

B. Phenotypes[a] by age group among individuals with ≥2 P/LP *SLC25A20* variants

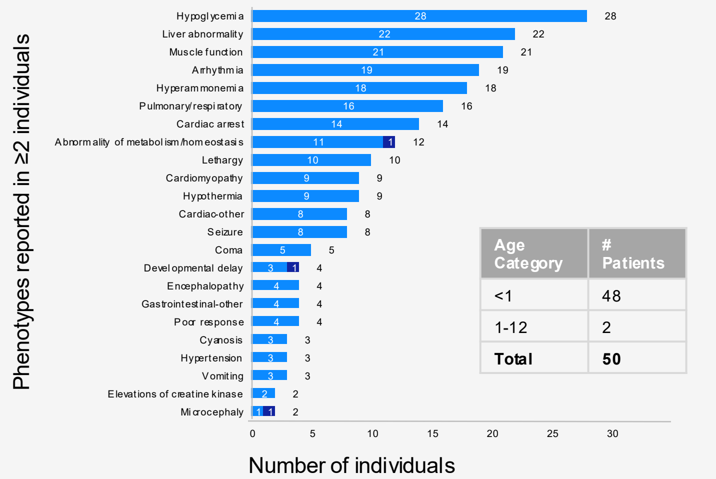

Age Category	# Patients
<1	48
1-12	2
**Total**	**50**

C. Phenotypes[b] by age group among individuals with ≥2 P/LP *CPT2* variants

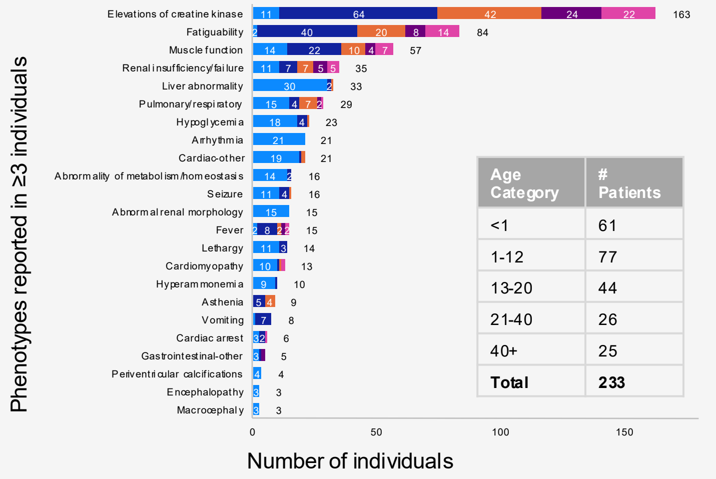

Age Category	# Patients
<1	61
1-12	77
13-20	44
21-40	26
40+	25
**Total**	**233**

D. Phenotypes[c] by age group among individuals with ≥2 P/LP *ACADVL* variants

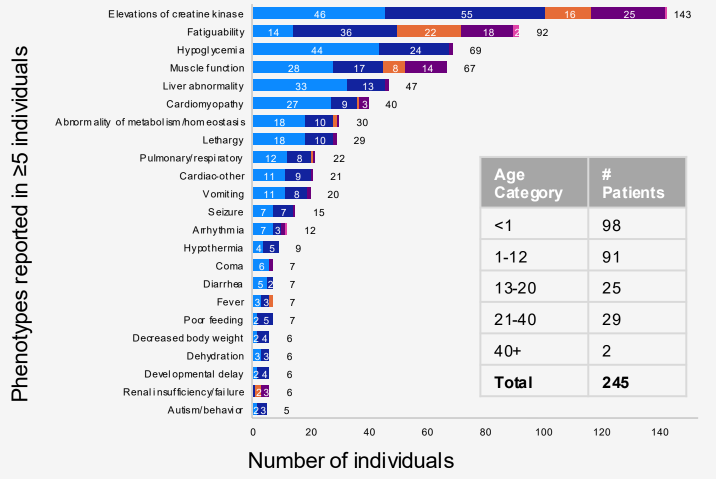

Age Category	# Patients
<1	98
1-12	91
13-20	25
21-40	29
40+	2
**Total**	**245**

E. Phenotypes[d] by age group among individuals with ≥2 P/LP *HADHA* variants

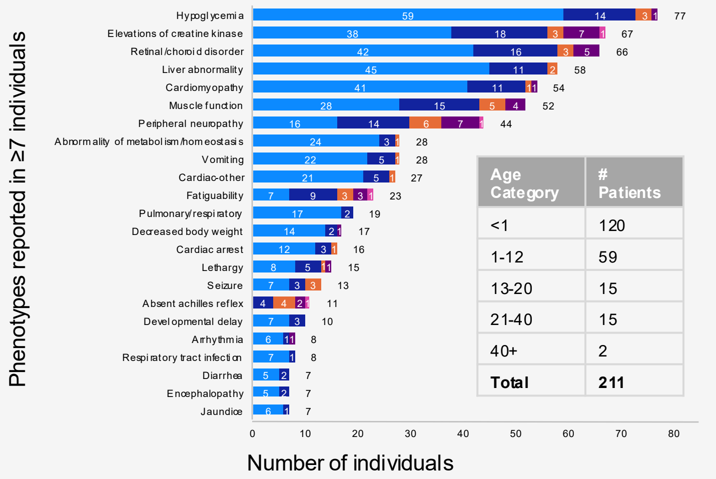

Age Category	# Patients
<1	120
1-12	59
13-20	15
21-40	15
40+	2
**Total**	**211**

F. Phenotypes[e] by age group among individuals with ≥2 P/LP *HADHB* variants

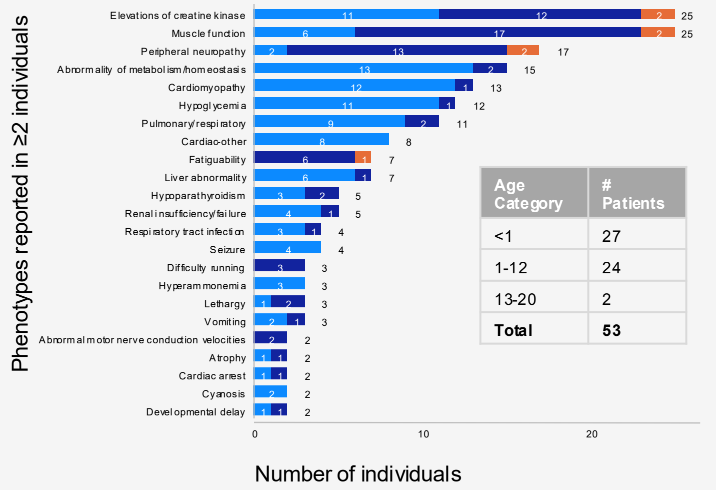

Age Category	# Patients
<1	27
1-12	24
13-20	2
**Total**	**53**

Figure S5. NBS blood spot acylcarnitine values as reported for (a) 241 patients with ≥2 P/LP variants and (b) 48 individuals with 2 variants, 1 or more VUS.

A

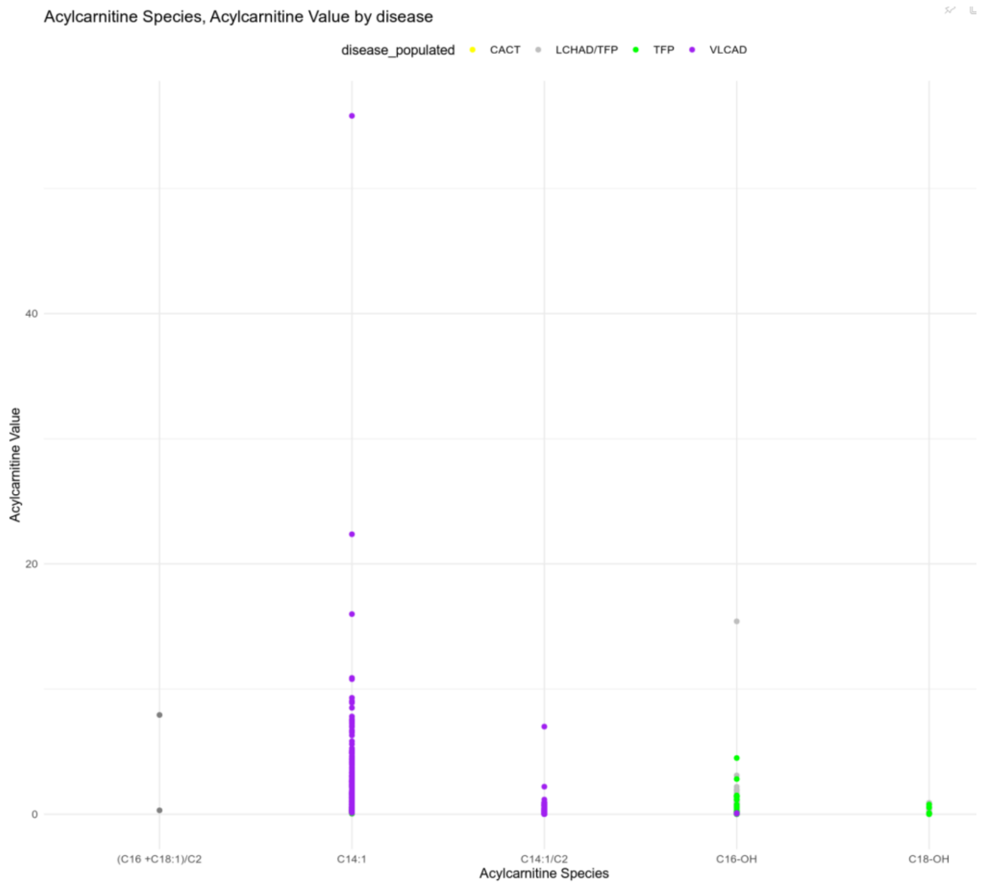

B

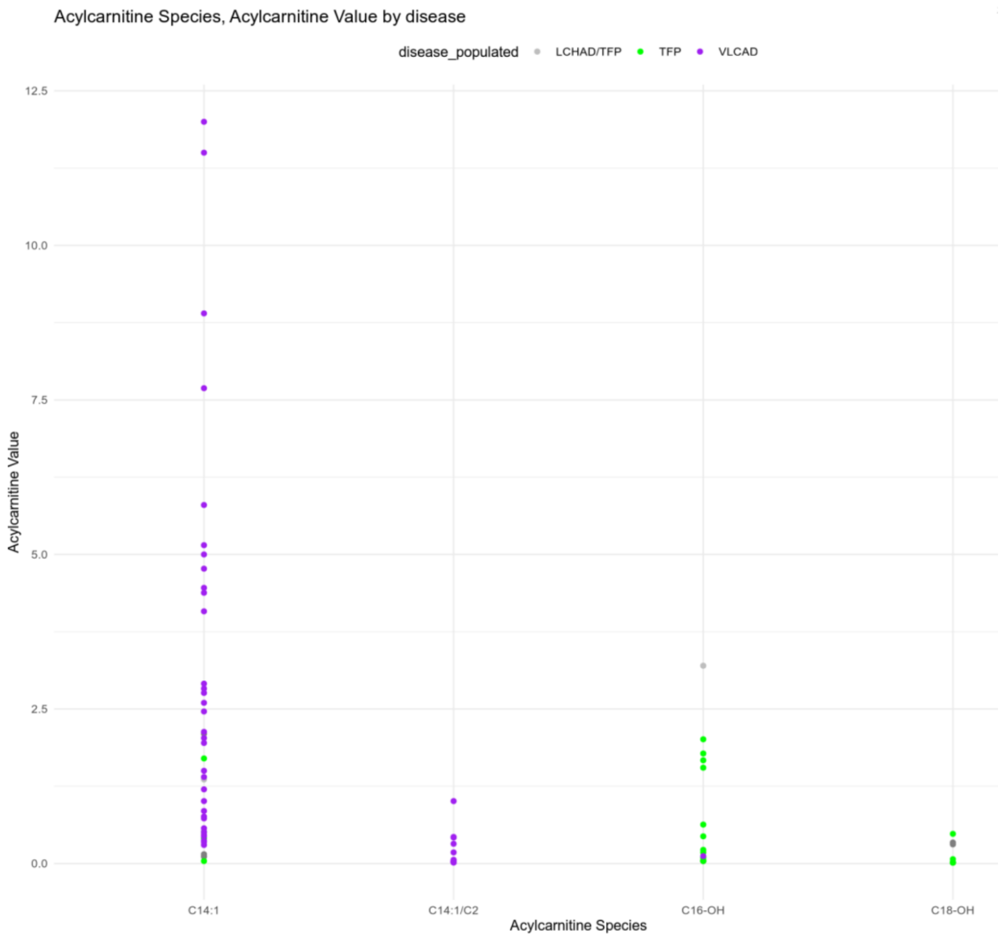

Figure S6. Cardiac manifestations occurred in 252 individuals with ≥2P/LP/VUS

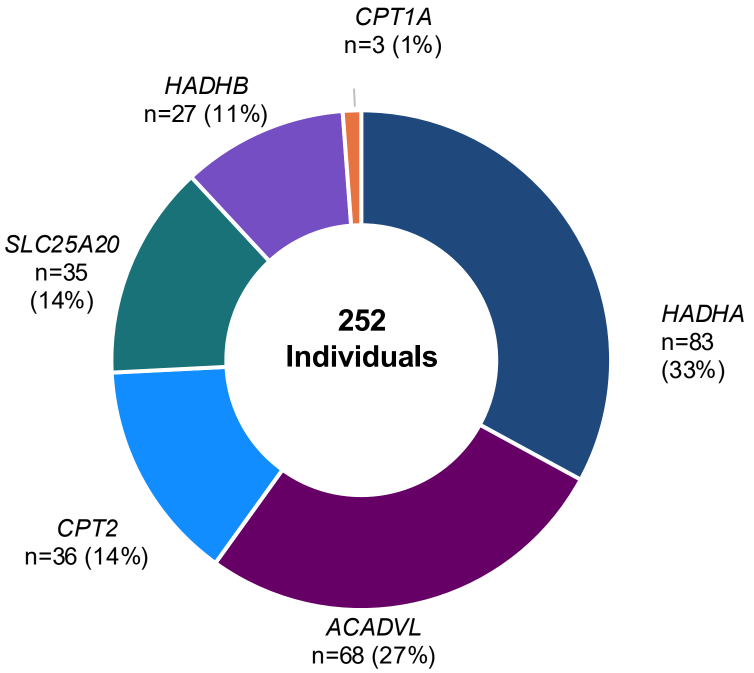

Supplement: Supplementary file 1 — Supporting information 1 Figure S1: Geographic location for 2350 individuals with ≥ 2 P/LP/VUS LC‐FAOD gene variants and geography reported∗. Figure S2: Frequency of LC‐FAOD subtype by age category for 1327 of 2372 (56%) individuals with ≥ 2 LP/P variants and age of diagnosis recorded. Figure S3: Abnormal newborn screening (NBS) results were reported for 1370 individuals (of 1399 reporting). Figure S4: Phenotypes by age group among individuals with ≥ 2 P/LP variants in an LC‐FAOD gene. Figure S5: NBS blood spot acylcarnitine values as reported for (a) 241 patients with ≥ 2 P/LP variants and (b) 48 individuals with 2 variants, 1 or more VUSs. Figure S6: Cardiac manifestations occurred in 252 individuals with ≥ 2 P/LP/VUS. [file HUMU-2026-6864813-s006.pdf]
